# Semiconductor Deposition via Laser Printing of a Bespoke Toner Containing Metal Xanthate Complexes

**DOI:** 10.1021/acsaenm.3c00709

**Published:** 2024-05-08

**Authors:** Paul D. McNaughter, Joshua Moore, Stephen G. Yeates, David J. Lewis

**Affiliations:** † Department of Chemistry, The University of Manchester, Oxford Road, Manchester M13 9PL, United Kingdom; ‡ Department of Materials, The University of Manchester, Oxford Road, Manchester M13 9PL, United Kingdom

**Keywords:** laser printing, xerography, toner, metal xanthate, semiconductor, metal chalcogenide

## Abstract

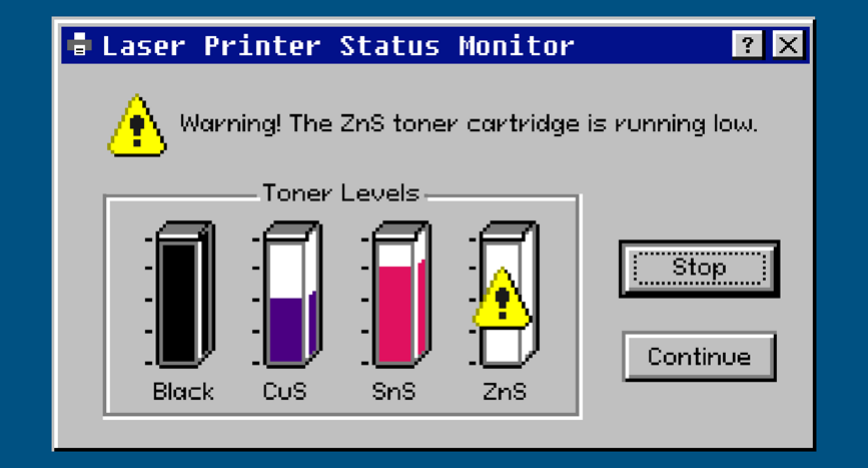

A methodology to use laser printing, a form of electrophotography, to print metal chalcogenide complexes on paper, is described. After fusing the toner to paper, a heating step is used to cause the printed metal xanthate complexes to thermolyze within the toner and form three target metal chalcogenides: CuS, SnS, and ZnS. To achieve this, we synthesize a poly(styrene-*co*-*n*-butyl acrylate) thermopolymer that emulates the thermal properties of a commercial toner and is also solution processable with the metal xanthate complexes used: [Zn(S_2_COEt)_2_], [Cu(S_2_COEt)·(PPh_3_)_2_], and [Sn(S_2_COEt)_2_]. We demonstrate through energy dispersive X-ray mapping that the toner is deposited following printing and that thermolysis of the metal xanthate complexes occurs in the fused toner, demonstrating the first example of laser printing of inorganic complexes and, in turn, semiconductors.

## 1 Introduction

Printed electronics offer an attractive route to the fabrication of high-volume and low-cost electronic circuitry.^1^ Initially dominated by organic semiconductors, in particular, semiconducting polymers, in the 1970s,^2,3^ printed electrics has since expanded to include inorganic conducting and semiconducting materials that are compatible with solution-based processing routes, e.g., inkjet printing and screen printing.^1,4−7^ All solution-based methods require careful design of the ink, but inkjet printing is particularly complex due to requirements for successful printing, i.e., achieving jetting, solubilizing or dispersing of the semiconductor (or precursors of), wetting the substrate, and drying correctly. When printing preformed semiconductor nanocrystals or two-dimensional materials, this can provide a challenging system to maintain colloidal stability while also meeting the requirements of printing.

Xerography, i.e., dry printing, was developed in 1959 for rapid sequential analogue copying and was later coupled with digital computers to form a modern-day laser printer. Elecrophotography, the process that underpins laser printing, has six steps that remain unchanged since the invention by C. F. Carlton in 1936: charge, expose, develop, transfer, fuse, and clean.^8,9^ During the exposure step, the charged photoreceptor drum is exposed to a laser beam that writes an electrostatic latent image to the drum surface. As the drum turns, the latent image passes a hopper containing a fine dust consisting of a pigment and a thermopolymer called toner.^10^ The toner is electrostatically attracted to the latent image on the drum, resulting in the latent image being covered in a layer of toner. The image is transferred from the drum to paper via a contact transfer roller and is fused using heat and pressure. Despite the increased printing speeds as compared to ink-based methods, xerography is under explored as a route to printing electronics. Xerography has been studied as a route to the printing of conductive paths of metals by the modification of toner to contain metallic particles or printing of coated metallic toner.^11−14^ The printing of semiconductors is unexplored and warrants development.

Metal chalcogenides are inorganic solid semiconductors that exhibit a wide range of band gap energies. The ability of chalcogens to bond with a wide variety of metals creates a wide selection of available band gaps with uses in photovoltaics and thermoelectrics.^15^ Metal sulfides have received the most attention due to the relative ease in the synthesis and the well-positioned band gaps for the harvesting of light, i.e., approximately 1.0 to 1.5 eV. The combination of band gaps near the visible range and Bohr radii in the order of nanometers also allows for the control of bandgap by use of quantum confinement.^16^ The expansion of 2D materials beyond graphene led to the exploration of layered transition metal dichalcogenides that can exist in monolayer forms.^17^ Narrow bandgap chalcogenides, e.g., Bi_2_Te_3_, have been used as thermoelectric materials and convert that to an electric current via the Seebeck effect.^18^

Metal xanthate complexes offer a synthetically convenient route to metal chalcogenide semiconductors due to the ease of synthesis, low thermolysis temperatures, and volatile byproducts. Thermolysis has been performed in solvents,^19−25^ in soft media,^26−30^ and by solventless thermolysis^24,31−41^ showing the versatility of metal xanthate complexes to form metal sulfides with control over size and composition. The ability to control the solubility of the complex through choice of alkyl group allows for solution processing techniques to be used.^32,34,39,41−43^ Consequently, control over metal xanthate solubility allows the potential for use of spin coating,^32,39−41,44^ drop casting,^45^ and the processing of the metal xanthate complex in combination with other dissolved molecules. In particular, the solution processing and cocasting with polymers allows for films with homogeneous mixtures of metal xanthate complex and the polymer of interest.^26−30^ This has been used as a route to the creation of bulk heterojunction thin films containing a semiconducting polymer and metal chalcogenide nanocrystals for light harvesting.^27,29,30^

When taking into account both the features of laser printing, which is a predominantly powder-based processing, and the production of metal chalcogenide semiconductors from metal xanthate powders using relatively low temperature processing in both the solid state and in polymers, it is clear that an opportunity exists to print metal chalcogenide semiconductors using a laser printer. We hypothesized that by the creation of bespoke toners containing metal xanthate complexes that are precursors for semiconductors by laser printing followed by a thermolysis step that we would be able to laser print a semiconductor infused toner for the first time. In this study, we therefore demonstrate a methodology for the printing of metal xanthate complexes in place of a traditional pigment. Building upon our previous studies of metal xanthate decomposition within polymers, we demonstrate the creation of a model thermopolymer toner embedded with metal xanthate complexes. Following laser printing of this bespoke metal xanthate infused toner, the complexes subsequently undergo thermolysis to form the respective crystalline metal chalcogenides of interest. This study demonstrates a method allowing the deposition of molecular complexes via laser printing with potential for further development with semiconducting polymer-based toners to make energy harvesting devices.

## 2 Experimental Section

### 2.1 Apparatus

Production of polymer and metal xanthate mixture fine powders was performed using a Fritsch P6 planetary ball mill equipped with a 12 mL zirconium oxide griding bowl and six 10 mm zirconium oxide milling balls. Laser printing was carried out using a Hewlett-Packard (HP) LaserJet P2055dn black and white printer with HP CE505A toner cartridges. Powder X-ray diffractograms were collected using a Phillips X’pert Pro with a CuKα source and a vertical ⊖–⊖ goniometer with an X’Celerator multistrip detector. Scanning electron micrographs and corresponding energy dispersive X-ray (EDX) maps were collected on a FEI Quanta 650 FEG-SEM equipped with an Oxford Instruments X-Max 50 EDX detector or an FEI Quanta 250 FEG-SEM equipped with an Oxford instruments X-Max 80 EDX detector. Elemental analysis, thermogravimetric analysis (TGA), and differential scanning calorimetry (DSC) were performed by the Department of Chemistry Microanalysis Laboratory at the University of Manchester using a Flash 200 Organic Elemental analyzer for CHN and S and a Thermo Scientific iCAP 6000 series ICP spectrometer for metal analysis. The heating of the printed paper was performed in a quartz tube under a nitrogen atmosphere in a Carbolite MTF 12/25/250 tube furnace. Raman microscopy was performed using a Renishaw inVia Reflex Raman spectrometer equipped with incident wavelengths of 325, 457, 488, 514, 633, and 785 nm and a 100× 0.85NA objective lens.

### 2.2 Materials

Styrene (≥99%, Sigma-Aldrich), *n*-butyl acrylate (≥99%, Sigma-Aldrich), acrylic acid (99%, Sigma-Aldrich), 1-dodecanethiol (≥98%, Sigma-Aldrich), 2,2′-azobis(2-methylpropionitrile) (98%, Sigma-Aldrich), 2,2′-azobis(2-methylbutyronitrile) (≥98%, Sigma-Aldrich), toluene (99.9%, Sigma-Aldrich), methanol (99.9%, Sigma-Aldrich), and A4 office paper (80 g m^–2^, white, Banner, #9150016) were used.

### 2.3 Synthesis of Poly(styrene-*co*-*n*-butyl acrylate)

Styrene (264.0 g, 2.53 mol), *n*-butyl acrylate (36.0 g, 0.28 mol), acrylic acid (6.0 g, 0.08 mol), 1-dodecanethiol (5.7 g, 0.3 mol), 2,2′-azobis(2-methylpropionitrile) (3.0 g, 0.02 mol), and 2,2′-azobis(2-methylbutyronitrile) (4.5 g, 0.02 mol) were combined and stirred until homogeneous. Toluene (450 mL) was heated to 80 °C in a reactor vessel fitted with an overhead stirrer. Once at temperature, the polymerization mixture was dissolved in the toluene and held at 80 °C with stirring for 25 h. The resultant polymer was precipitated into an excess of methanol, isolated, and dried (60.0 g, 20%). (Found: C, 86.89; H, 8.09. Calc. for [poly(styrene-*co*-*n*-butyl acrylate) with a styrene:*n*-butyl acrylate of 0.14:0.86]: C, 87.80; H, 8.02%) ^1^H NMR (500 MHz, CDCl_3_) δ 7.68–5.80 (5H, br m, C_6_H_5_), 4.30–3.27 (2H, br m, CH_2_), 2.76–0.4 (5H, br m, CH_2_CH_3_) ν_max_/cm^–1^ 3023 (Aryl-H stretch), 2921 (C–H stretch), 1726 (C=O stretch), 1492 (C–H bend), 1452 (C–H bend), and 1154 (C–O stretch).

### 2.4 Toner Cartridge Emptying and Refilling

The commercial toner cartridge is emptied by drilling two holes in the compartment storing the toner, see Figure S1 for the drilling locations. The contained toner, which shall now be referred to as the commercial toner, is collected. Residual commercial toner is removed from the cartridge by the repeated use of compressed air until the toner stops leaving the cartridge. Refilling of the cartridge is achieved by pouring the desired toner mixture into one of the holes. After addition, the holes are sealed and the cartridge wrapped in aluminum foil. The cartridge is inverted along the long axis ten times and placed on an orbital shaker at 300 rpm for 1 h.

### 2.5 Toner Substitute Containing Metal Xanthate Complexes

The synthetic procedures of [Cu(S_2_COEt)·(PPh_3_)_2_], [Zn(S_2_COEt)_2_], and [Sn(S_2_COEt)_2_] are provided in the Supporting Information and are modified from previous reports.^31,46−48^ In a typical milling, 0.03 mol of a xanthate complex is added to 1.8 g of poly(styrene-*co*-*n*-butyl acrylate). The mixture of powders is ground in a pestle and mortar until a uniform fine powder is made. 1 g of this mixture is placed in a 12 mL zirconium oxide grinding bowl with six 10 mm zirconium oxide milling balls. The mixture is milled for 99 repetitions of milling at 250 rpm for 1 min and resting for 3 min in order to avoid the buildup of heat. The powder is collected and stored at −20 °C. 0.5 g portion of the precursor-polymer milled powder is added to 0.5 g of the commercial toner and shaken until uniformly mixed.

### 2.6 Laser Printing

A test page containing the word “TEST” in a sans serif font (font Calibri at size 72) and two solid rectangles (13.5 cm × 2.5 cm) and nine solid squares (2.5 cm × 2.5 cm) is used for all printing tests. The test page is included as the Supporting Information. After each page is printed, the cartridge is removed from the printer and inverted ten times. Three test pages were printed, and a section of the second page is sampled for the analysis shown.

### 2.7 Thermolysis of Printed Metal Xanthate Complexes

A 1.5 cm × 2.5 cm section of printed paper was removed from the sample page. This was placed on a flat glass slide and inserted into a 2.5 cm diameter quartz tube and was connected to a Schlenk line. The atmosphere was replaced with nitrogen by three cycles of evacuation and refilling with nitrogen. The sample was heated to the target temperature and held at that temperature for the desired time. The tube was removed from the furnace and allowed to cool in air before the sample.

## 3 Results and Discussion

### 3.1 Polymer Properties

Examination of the aryl and the O–CH_2_ envelopes in the polymer ^1^H NMR spectrum gave a ratio of styrene:*n*-butyl acrylate of 0.14:0.86 confirming the composition as required for the target *T*_g_. The thermal properties of the poly(styrene-*co*-*n*-butyl acrylate) were examined using TGA and DSC to allow comparison to the commercial HP toner (Figure S2). Poly(styrene-*co*-*n*-butyl acrylate) begins to lose weight at approximately 300 °C, and a glass transition temperature, *T*_g_, is observed at 77 °C. A low *T*_g_ is ideal for the flash heating used to fuse the laser toner to paper indicating the synthesized poly(styrene-*co*-*n*-butyl acrylate) would be suitable as a model polymer for laser printing.

### 3.2 Metal Xanthate Containing Toner Production

**Figure 1:**
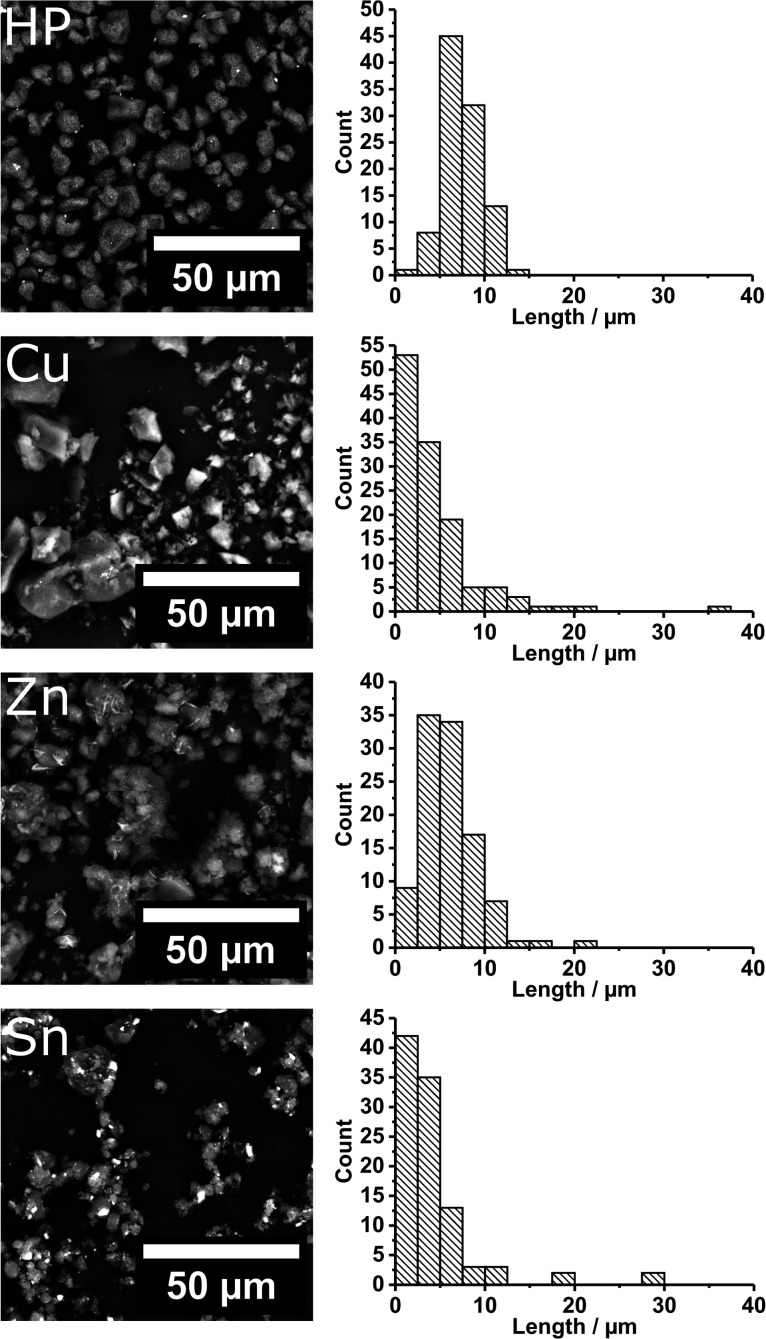
SEM micrographs (left) and corresponding size distributions (right) for the commercial HP toner, [Cu(S_2_COEt)·(PPh_3_)_2_]-containing toner, [Zn(S_2_COEt)_2_]-containing toner, and [Sn(S_2_COEt)_2_]-containing toner.

To attain a target size for our bespoke precursor containing toner particles, the commercial toner was examined using SEM (Figure 1, top). The commercial toner displayed randomly shaped particles of approximately 7.5 μm in diameter and a narrow size distribution.

A common route for the manufacture of toner is extrusion and pulverization. The substitution of pigment for metal xanthate complexes eliminates this route, as the high temperatures required in extrusion would cause the complexes to thermolyze prematurely. Ball milling offers the potential to make fine powders while controlling the sample temperature by control of a range of parameters, e.g., ball to vesicle to milled material ratio, speed of rotation, duration of milling, pulsed milling, etc. When ball milling a thermopolymer and metal xanthate complexes, a temperature too high can result in either (i) exceeding the glass transition temperature of the polymer causing the powder to fuse or (ii) the premature thermolysis of the metal xanthate complexes. By milling at a low rate, i.e., 250 rpm, and in bursts of 1 min followed by a rest of 3 min, we found the milling process could reduce the size of the metal xanthate-poly(styrene-*co*-*n*-butyl acrylate) particles without heating the sample detrimentally (Figure 1 and Table S1). The mean size of the ball milled polymer/metal xanthate particles is typically slightly smaller than the commercial toner at 4.46 to 6.07 μm. The polymer/metal xanthate particle size distribution was found to be consistently wider with standard deviations of 3.13 to 4.75 μm. We noted that the orbital ball milling produces wider size distributions than pulverization due to the random nature of collisions between the ball, wall, and milled material. The milled polymer/metal xanthate particle diffractograms displayed reflections characteristic of the unreacted metal xanthate complexes and no reflections that could be attributed to the corresponding metal sulfide phases indicating the metal xanthate complexes did not thermolyze during milling (Figure S3).

**Figure 2:**
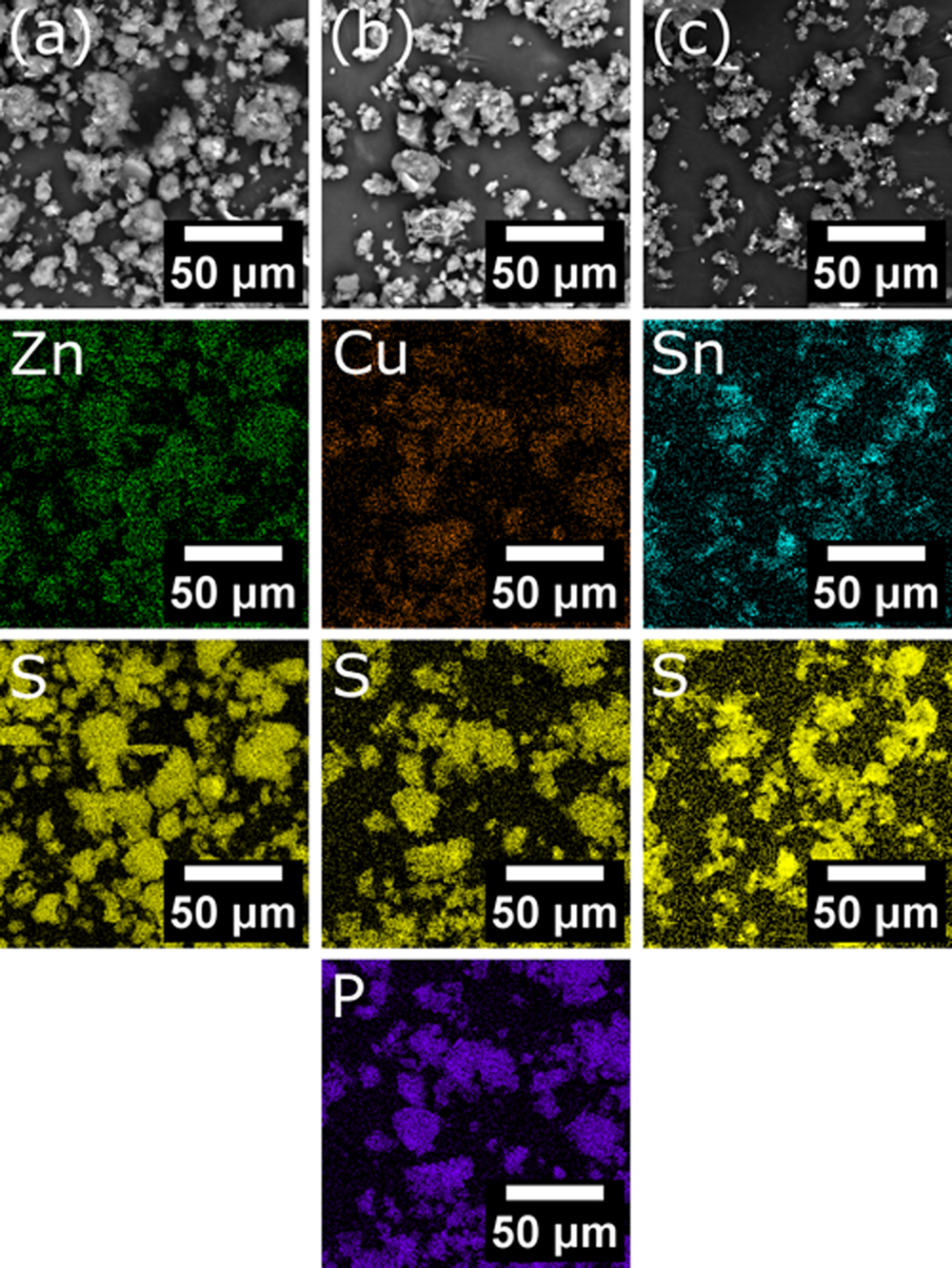
EDX maps showing the distribution of elements indicating the location of the metal xanthates used in the milled mixture of poly(styrene-*co*-*n*-butyl acrylate) with [Zn(S_2_COEt)_2_] column (a), [Cu(S_2_COEt)·(PPh_3_)_2_] column (b), and [Sn(S_2_COEt)_2_] column (c).

To maximize a uniform distribution of metal xanthate within the polymer, prior to ball milling the poly(styrene-*co*-*n*-butyl acrylate) and respective metal xanthate complex were mixed by dissolving in chloroform and the subsequent removal of the solvent. The resulting mixture of poly(styrene-*co*-*n*-butyl acrylate) and a metal xanthate complex was uniform in appearance. Following ball milling, the poly(styrene-*co*-*n*-butyl acrylate) and metal xanthate powders were investigated by EDX mapping to determine the degree of mixing (Figure 2). In all of the powders, the metal of interest and sulfur was observed where the polymer particles were found. In the case of [Cu(S_2_COEt)·(PPh_3_)_2_], phosphorus was also colocalized with the copper and sulfur further indicating that the complex was uniformly distributed across the polymer particles. The ratio of EDX quantifications confirmed that the complexes remained intact following milling, Table S2.

Our previous publications investigating the formation of metal xanthate containing polystyrene films via spin coating were used as a starting point in the design of our metal xanthate containing toner particles.^26 ,28 ,49^ A molar ratio of metal xanthate to polymer repeating unit of 6:1 was used for initial attempts, i.e., 0.1 mol of metal xanthate and 1.8 g, and resulted in incomplete printing coverage of the toner mixtures explored. The amount of metal xanthate relative to the polymer was lowered until complete printing coverage was observed when using 0.3 mol of metal xanthate and 1.8 g of polymer.

### 3.3 Thermolysis of Metal Xanthate Complexes within Polymer

**Figure 3:**
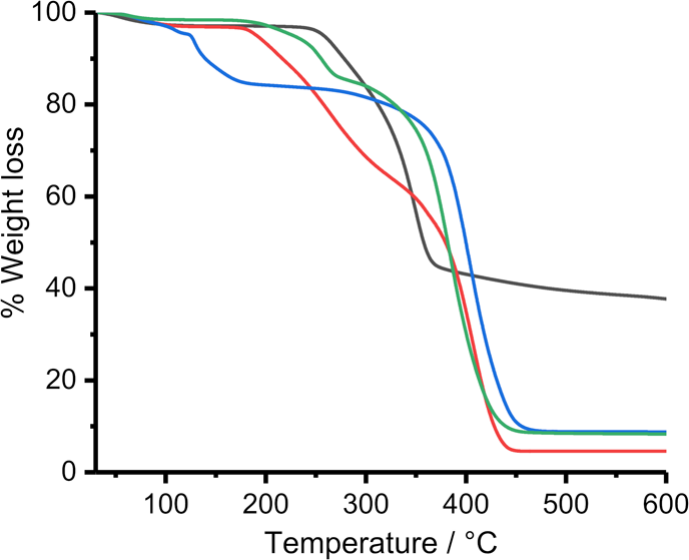
Thermogravimetric analysis profiles for printer paper (black) and metal xanthate complexes mixed poly(styrene-*co*-*n*-butyl acrylate) with profiles for [Zn(S_2_COEt)_2_] (blue), [Cu(S_2_COEt)·(PPh_3_)_2_] (red), and [Sn(S_2_COEt)_2_] (green).

The thermal properties of the printer paper and the milled mixtures of metal xanthate and poly(styrene-*co*-*n*-butyl acrylate) were observed by TGA (Figure 3). Printer paper begins to lose weight when heated beyond 250 °C (Figure 3, black). All three metal xanthate and poly(styrene-*co*-*n*-butyl acrylate) milled powders begin to lose weight by 200 °C although their TGA profiles continue to have features beyond the significant weight loss of paper, i.e., above 350 °C, and below the beginning of the thermal breakdown of the polymer at 300 °C (Figure S2). The lower breakdown temperatures of the metal xanthate complexes relative to paper mean that the metal xanthate complexes used can thermolyze to form metal chalcogenides before the paper substrate is damaged by heating.

**Figure 4:**
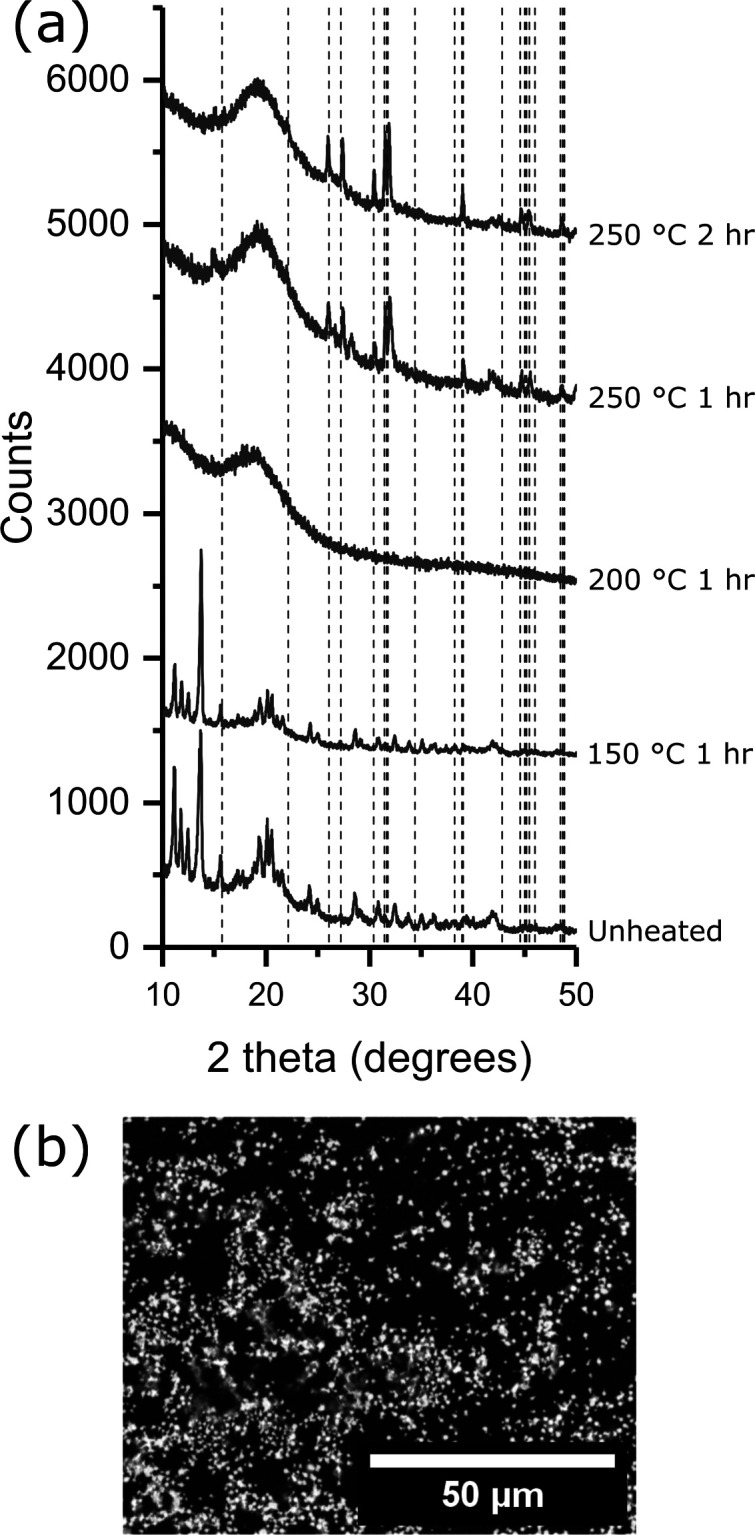
(a) XRD diffractograms of ball milled poly(styrene-*co*-*n*-butyl acrylate) with [Sn(S_2_COEt)_2_] following heating under nitrogen. The dashed lines represent the 2 theta values for SnS ICSD #106030. (b) SEM micrograph of SnS particles in poly(styrene-*co*-*n*-butyl acrylate) following heating at 250 °C for 2 h.

To optimize the time and temperature required for the postprinting conversion step, the metal xanthate-poly(styrene-*co*-*n*-butyl acrylate) milled powders were heated under nitrogen. The progress of the thermolysis was assessed by powder X-ray diffraction and was used to confirm the formation of the target metal chalcogenide semiconductors in the bespoke toners (Figures 4, S4, and S5). Prior to heating, all unheated bespoke toners displayed reflections characteristic of the unreacted metal xanthate complexes and no reflections that could be attributed to the corresponding metal sulfide phases indicating the metal xanthate complexes did not thermolyze during milling. In the case of the [Zn(S_2_COEt)_2_]-poly(styrene-*co*-*n*-butyl acrylate) milled powders, reflections from [Zn(S_2_COEt)_2_] disappeared after heating at 150 °C for 1 h and broad reflections characteristic of small ZnS crystallites emerged corresponding to sphalerite, ISCD #108733 (Figure S4). The broad reflections became more pronounced with higher heating temperatures and longer times. [Cu(S_2_COEt)·(PPh_3_)_2_] showed reflections from the precursor at 150 °C which disappeared when heating at 200 °C for 1 h and the emergence of peaks from chalcocite Cu_2_S, ISCD #106030 (Figure S5). When heating at 250 °C for 2 h, an increased number of well-resolved reflections were observed, suggesting an increased quantity of material was made. Reflections from [Sn(S_2_COEt)_2_] observed after 150 °C for 1 h. After heating at 200 °C for 1 h, an amorphous mixture was produced with no reflections observable, Figure 4a. Heating at 250 °C for 1 h resulted in a mixture of SnS and with a minor phase of SnS_2_ present. The minor phase of SnS_2_ was eliminated by heating at 250 °C for 2 h producing only SnS, Figure 4a. An SEM micrograph of the SnS particles formed shows that they are randomly distributed within the polymer and unaggregated, Figure 4b, and this is also observed with the CuS and ZnS particles (Figures S4 and S5).

### 3.4 Laser Printing of Bespoke Metal Xanthate Containing Toner

The milled mixtures of metal xanthate complexes and poly(styrene-*co*-*n*-butyl acrylate) were loaded into emptied toner cartridges, shaken for 2 h prior to printing. It was observed that no discernible amount of the metal xanthate toner was found on the paper in the regions of printing. This is not entirely unexpected as the composition of our simplified toner does not contain all of the main components of a commercial toner, i.e., flow enhancing waxes and charging agents. It should also be noted that the toner is coengineered with the cartridge to match the properties of the toner used. This is unlike inkjet printing where there is greater conformity in nozzles and jetting mechanisms, and ink formulations are adapted to work with a range of standard printing conditions.

**Scheme 1:**
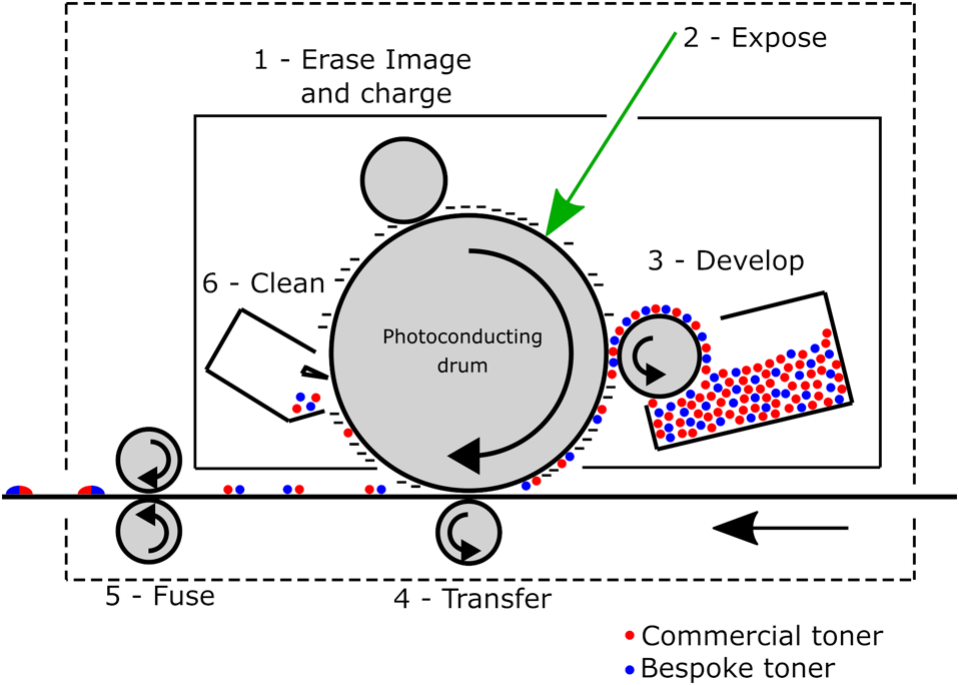
Laser Printing of a Mixture of the Commercial Toner (blue circles) and the Bespoke Metal Xanthate Containing Toner (red circles) with the Six Stages of Electrophotography Shown

To encourage the flow of material from the toner cartridge, we mixed our metal xanthate embedded poly(styrene-*co*-*n*-butyl acrylate) powders with the commercial toner extracted from the cartridges 50:50 by weight. This mixture was subsequently shaken to make a uniform mixture of the commercial toner and the metal xanthate containing polymer powder. The mixture was loaded into a cartridge and shaken for 2 h prior to printing (Scheme 1).

**Figure 5:**
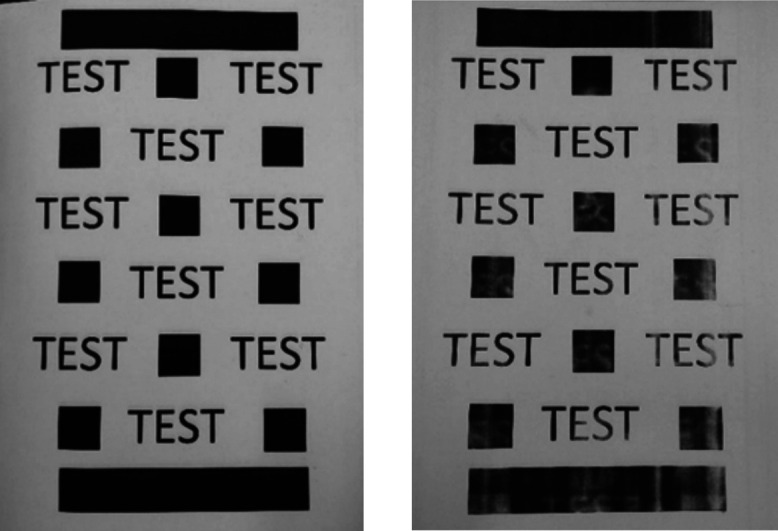
An example of the test page created to print the bespoke metal xanthate containing toners. (Left) Printed with commercial HP toner, and (right) a typical page printed using a mixture of bespoke toner and HP toner.

**Figure 6:**
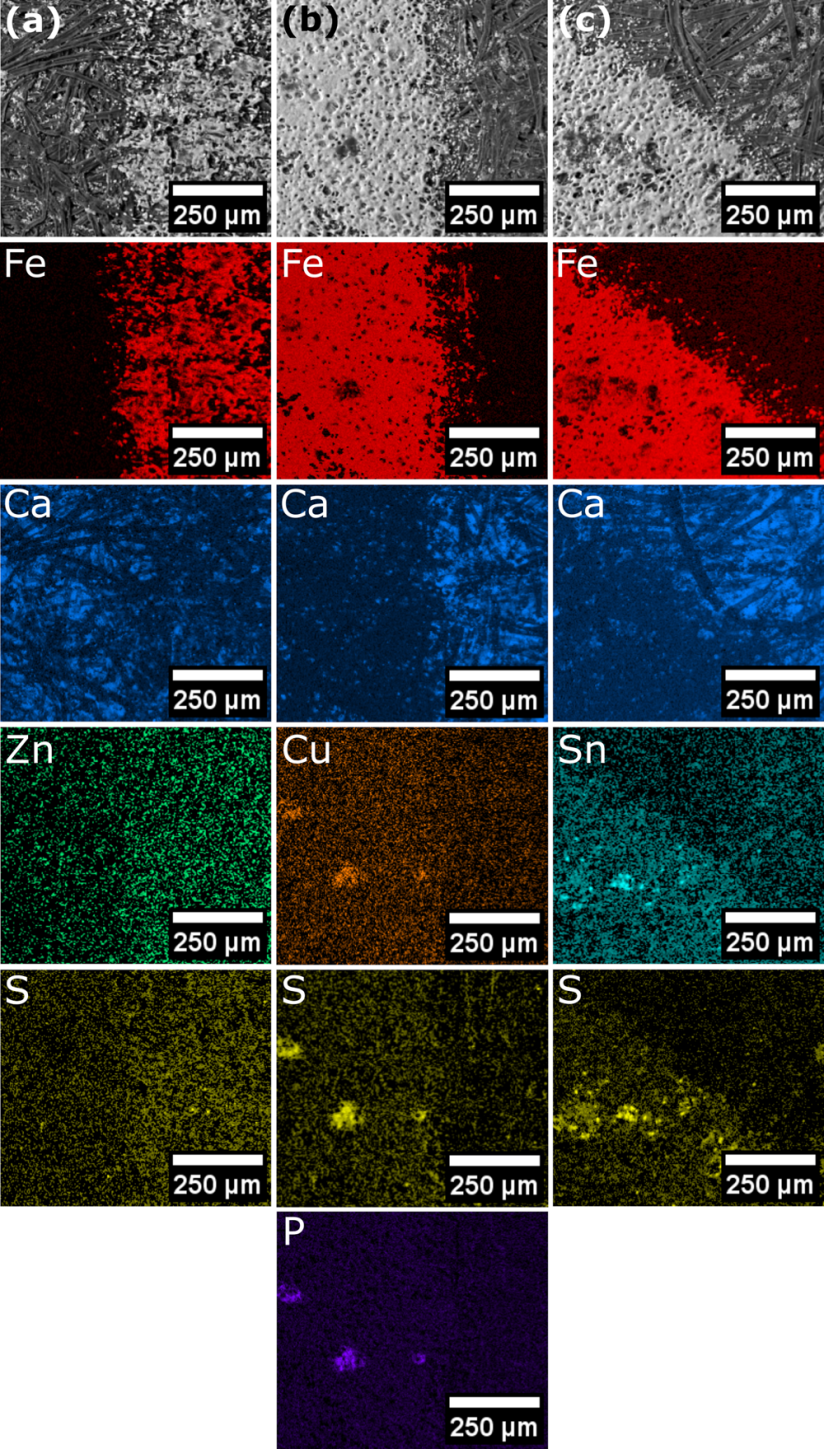
EDX maps of metal xanthate containing poly(styrene-*co*-*n*-butyl acrylate) printed with HP toner where the column (a) [Zn(S_2_COEt)_2_], column (b) [Cu(S_2_COEt)·(PPh_3_)_2_], and column (c) [Sn(S_2_COEt)_2_].

A representative example of a printed page is shown in Figure 5 (right). The representative sample displays minor ghosting observed as the absence of toner in a printed region in the shape of a previously printed object. This is expected to be a consequence of the differences in size distributions of the bespoke toner compared to those of the commercial toner. Bespoke toner particles smaller than the commercial toner may evade the cleaning step of electrophotography. For example, if the toner dimensions were too small the common method of cleaning the photoconductive drum via a doctor blade would not remove the toner smaller than the gap between drum and blade. Another consideration is our simplified toner not containing flow and charging agents specific to this model of printer. We do not believe this is a hot offset contamination of the fuser roll, i.e., melted toner on the fuser roll, due to the lack of printed toner in unwanted regions. Following printing on paper, the fused toner was imaged by SEM and EDX spectroscopic mapping (Figure 6). The edges of a printed rectangle were investigated to allow comparison among a large continuously printed area, printed text, and unprinted paper. The commercial toner contains Fe_3_O_4_, and this allows Fe (displayed as red) to act as an indicator of the location of the mixed metal xanthate–HP toner. The paper used in this study contains CaCO_3_ as a filler material to add bulk and to also make paper appear a brighter white to improve the opacity and in turn the ability to read text.^50,51^ This allows Ca (displayed as blue) to be followed by EDX mapping as an indication of unprinted paper or where holes in fused toner coverage occur. For all samples observed, the Fe and Ca signals show a clear demarcation at the edge of where printing has occurred (Figure 6).

The location of the metal xanthate complexes can also be determined following the X-ray emission profiles for copper, tin, zinc, and sulfur (Figures 6, S6, S7, and S8). In all cases, sulfur colocalizes with the corresponding metal of the metal xanthate complex used. In the case of [Cu(S_2_COEt)·(PPh_3_)_2_], phosphorus also colocalizes with copper and sulfur in further agreement of the location of the metal xanthate complexes. The size distribution of the metal xanthate poly(styrene-*co*-*n*-butyl acrylate) is broader, and there are polymer particles larger than that of the commercial toner. When printing [Sn(S_2_COEt)_2_] and [Cu(S_2_COEt)·(PPh_3_)_2_], the printed area displays regions of partially fused larger toner particles and smooth regions where smaller toner particles have successfully fused. In the case of [Zn(S_2_COEt)_2_], the distribution of Zn is more uniform. The mixed degree of fusing can be attributed to differences in the thermal properties of the poly(styrene-*co*-*n*-butyl acrylate) thermopolymer used as compared with the unknown thermopolymer used in the commercial toner and the differences in size distribution. The fusing process using temperatures between 200 and 400 °C and a sheet of paper passes across the fuser in under a second. Commercial toners are coengineered to the fuser temperature used. Our poly(styrene-*co*-*n*-butyl acrylate) is a good approximation of a typical thermopolymer used in toner but is not coengineered with the fuser used, causing mixed fusing results. Line scans to observe the change from unprinted to printed areas were used to examine the location of elements taking advantage of the superior signal-to-noise achievable in realistic time scales, and the line scans confirmed the colocalizations observed in the maps (Figures S6–S8).

**Figure 7:**
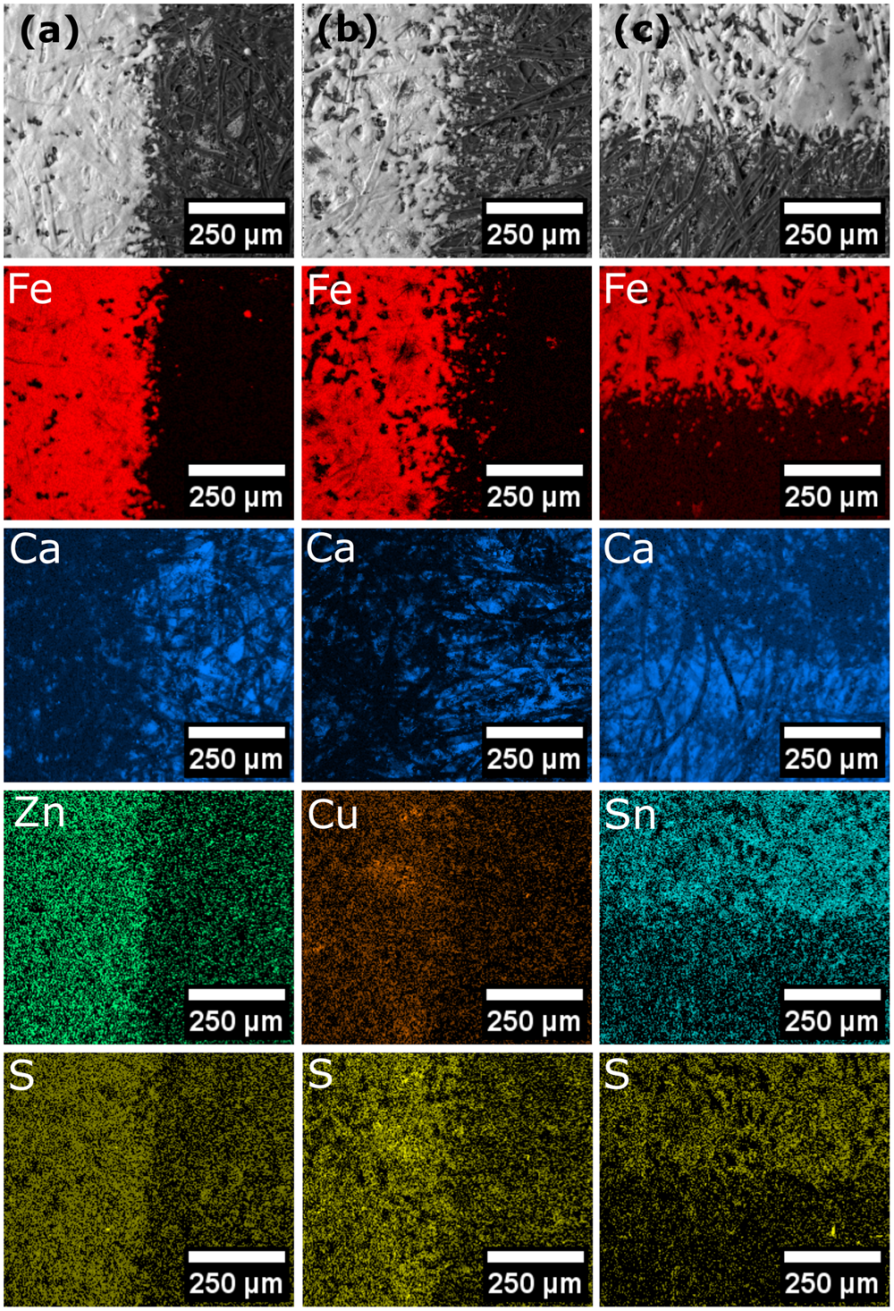
Column (a) [Zn(S_2_COEt)_2_], column (b) [Cu(S_2_COEt)·(PPh_3_)_2_], and column (c) [Sn(S_2_COEt)_2_] printed with HP toner following heating at 250 °C for 2 h.

Following heating at 250 °C for 2 h, the printed paper was also imaged by SEM with EDX spectroscopic analysis (Figures 7 and S9). The locations of the elements of interest continue to coincide with the location of the printed toner; i.e., no elements have migrated from a printed region to an unprinted region. An improvement in the distribution of Cu and Sn is observed in the EDX maps following heating the printed samples. This is attributed to the completion of fusing of larger milled toner particles in the postprinting heating step. In the case of Sn, Zn, and Cu, all show a clearer demarcation with the edge of the toner than before heating suggesting a better distribution of the metals through the printed regions. After heating the [Cu(S_2_COEt)·(PPh_3_)_2_] toner, the phosphorus signal is no longer observable in the map sum spectrum indicating successful precursor breakdown and the evaporation of triphenylphosphine (Figure S9). EDX spectra of the printed and unprinted regions show an absence of Cu where the paper is unprinted and Cu where the toner has fused (Figures S6 and S9). Line scans were performed and confirmed the location of the Cu, Zn and Sn in the printed regions (Figures S10 −S12).

**Figure 8:**
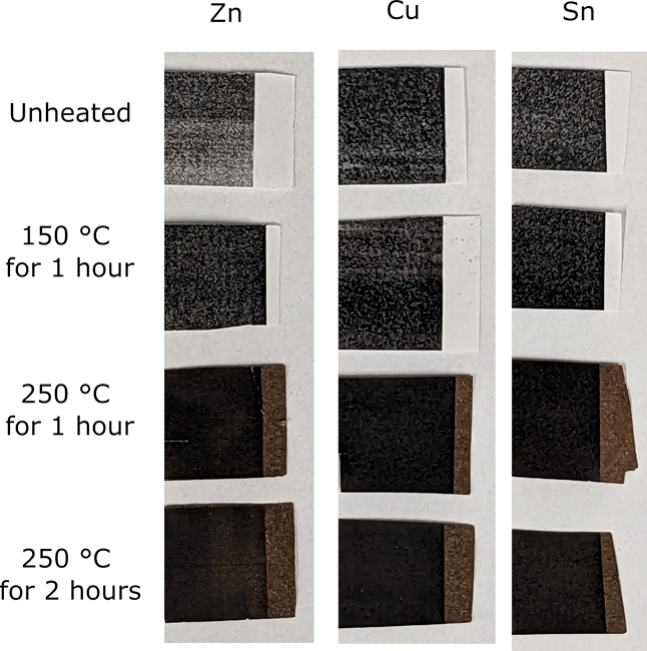
Photographs of paper printed with the metal xanthate containing poly(styrene-*co*-*n*-butyl acrylate) toner mixed with a commercial HP toner.

Figure 8 shows paper samples following printing and after heating at the used temperatures and times. At 250 °C, the paper changes in appearance from white to brown. Wood-based papers turn brown over time due to the residual lignin content discoloring, whereas paper using cellulosic fibers sourced from cotton, flax, etc., turns brown to a lesser extent. Using higher temperatures is likely to make the browning process happen at a quicker rate.

The paper printed with metal xanthates and heated to 250 °C was examined by powder XRD to observe the metal chalcogenide inorganic phases (Figure S13). Despite witnessing the presence of the metals and sulfur by EDX mapping, the inorganic phases were not observed in the diffractograms due to the very small amount of material and abundance of competing diffracting material; CaCO_3_ of the paper and Fe_3_O_4_ from the commercial toner are readily observed in all the diffractograms for the different metal xanthate complexes used. This is likely a consequence of the volume fraction of each inorganic phase present and the consequent scattering intensity. The metal chalcogenide is far lower in relative quantity, and the reflections do not generate a large signal.

In an effort to observe the metal chalcogenide phases, Raman was also employed. Broad luminescence features were observed from paper, the commercial toner, and poly(styrene-*co*-*n*-butyl acylate) that spanned the visible and infrared spectral regions, Figure S14. The observed luminescence dominated the collected Raman spectra. Incident wavelengths of 325, 457, 488, 514, 633, and 785 nm were explored to minimize the contribution from the luminescence. 325 and 785 nm were used to avoid the most intense regions of the luminescence profiles but no Raman signals in the region expected for the metal chalcogenides were observed.^52^ The combined luminescence from the organic polymers and potentially the metal chalcogenides prevented observation of Raman signals previously observed for the controls, Figures S15, S16, and Table S3. Raman spectra for the heated metal xanthate-poly(styrene-*co*-*n*-butyl acrylate) mixtures and the printed bespoke toner are provided in Figures S17–S22.

## 4 Conclusion

A method for the direct writing of molecular complexes using laser printing and the subsequent formation of semiconductors with applications in energy harvesting has been demonstrated. A model toner system approximating commercial toner containing semiconductor precursor molecules in place of a pigment has been produced using metal xanthate complexes, [Zn(S_2_COEt)_2_], [Cu(S_2_COEt)·(PPh_3_)_2_], and [Sn(S_2_COEt)_2_], and a poly(styrene-*co*-*n*-butyl acrylate) thermopolymer. This was mixed with the commercial toner and printed via laser printing to deposit the metal xanthate complexes on standard office printer paper. After printing, the metal xanthate complexes have been thermolyzed by heating the printed paper to produce the semiconductor materials. The printed regions were examined by EDX spectroscopic mapping whereby uniform distributions of the semiconductor elements were observed in the printed regions. This solid-state approach to the printing of semiconductors is a new paradigm that may be exploited potentially using a range of currently existing “bottom-up” materials chemistry approaches and as such will be of interest to many for the direct deposition of useful materials, and is complementary to existing processes such as inkjet printing. The development of semiconducting thermopolymer-based toners would allow this model system to be expanded to develop a route to the direct writing of energy harvesting devices.

## Supporting Information Available

The synthetic procedures for the metal xanthate complexes; additional characterization for the toners produced, the printed toners, and the printed toners following heating; additional materials characterization includes thermal gravimetric analysis, differential scanning calorimetry, powder X-ray diffractograms, secondary electron micrographs, energy dispersive X-ray spectroscopic data, Raman spectra, and luminescence spectra (PDF)
The printing test page (PDF)
