# Supplementary material for: Semiconductor Deposition via Laser Printing of a Bespoke Toner Containing Metal Xanthate Complexes
Source: ACS Appl Eng Mater. 2024 May 8;2(5):1225–33. doi: 10.1021/acsaenm.3c00709 (PMC11129185; doi:10.1021/acsaenm.3c00709)
Supplement: Supplementary file 1 — em3c00709_si_001.pdf [file em3c00709_si_001.pdf]

Supporting Information

Semiconductor deposition *via* laser printing of a bespoke toner containing metal xanthate complexes

Paul D. McNaughter,[a†]* *Joshua Moore,*[a] *Stephen G. Yeates*[a] *and David J. Lewis*[b]*

[a] Department of Chemistry, The University of Manchester, Oxford Road, Manchester M13 9PL United Kingdom

[b] Department of Materials, The University of Manchester, Oxford Road, Manchester M13 9PL United Kingdom

Materials

Potassium ethylxanthogenate (96%, Sigma-Aldrich), triphenylphosphine (99 %, Sigma-Aldrich), copper(I) chloride (≥99 %, Sigma-Aldrich), zinc(II) acetate (≥98 %, Sigma-Aldrich), tin(II) chloride (≥99.99 %, Sigma-Aldrich), chloroform (≥99.5 %, Sigma- Aldrich), magnesium sulfate (≥98 %, Sigma-Aldrich) and dichloromethane (≥99.8 %, Sigma-Aldrich). Synthesis of the metal xanthate complexes was performed under Schlenk conditions under nitrogen unless otherwise stated.

Synthesis of zinc(II)ethyl xanthate, [Zn(S$_2$COEt)$_2$]

The procedure used is adapted from the method of Kociok-Köhn *et al.*.[1] Zinc(II) acetate (0.03 mol, 5.96 g) in distilled water (100 ml) was added dropwise to a stirred solution of potassium ethylxanthogenate (0.06 mol, 10.42 g) in distilled water (200 ml) in air. Upon addition, a colourless precipitate was formed instantly. Once addition was complete the reaction mixture was stirred for a further 30 minutes. The colourless precipitate was collected by vacuum filtration and was washed with distilled water (200 ml) and methanol (200 ml) before being dried in air resulting in a colourless powder collected (7.16 g, 71.6%). (Found: C, 23.7; H, 3.1;

S, 41.5; Zn, 21.8. Calc. for [$Zn(S_2COEt)_2$]: C, 23.4; H, 3.3; S, 41.5; Zn, 21.2%) ^{1}H NMR (500 MHz, MeOD) δ 4.47 (2 H, q, CH_2), 1.39 (3 H, t, CH_3). ^{13}C NMR (126 MHz, MeOD) δ 230.73, 73.44, 14.32. ν_{max}/cm^{-1} 2992 (s, CH), 1442 (bend asymmetrical, CH), 1366 (b, symmetrical CH), 1190 (s, C-O-C), 1120 (s, CO), 1020 (C=S).

Synthesis of copper(I) ethylxanthate.tripheylphosphine, [$Cu(S_2COEt).(PPh_3)_2$]

The procedure used is adapted from the methods of Kumar *et al.* and Cariati *et al.*.[2,3] Copper(I) chloride (2.988 g, 0.03 mol) and triphenylphosphine (15.89 g, 0.06 mol) were refluxed in dichloromethane (250 ml) for 12 hours and was allowed to cool to room temperature. Potassium ethyl xanthogenate (4.85 g, 0.03 mol) was added to the reaction mixture and was stirred at room temperature for 5 hours and a pale green precipitate formed. The reaction mixture was exposed to air and the volume of dichloromethane was reduced till a precipitate formed. The pale green solid was dissolved in a minimum amount of warm dichloromethane and was cooled to -20 ˚C and left overnight to form pale green crystals (14.21 g, 64.0%). (Found: C, 66.4; H, 5.0; S, 8.9; Cu, 8.8; P, 8.3. Calc. for [$Cu(S_2COEt).(PPh_3)_2$]: C, 67.1; H, 5.1; S, 8.7; Cu, 8.7; P, 8.3%) ^{1}H NMR (500 MHz, $CDCl_3$) δ 7.34-7.19 (15 H, m, C_6H_5), 4.41 (2 H, q, CH_2), 1.35 (3 H, t, CH_3). ^{13}C NMR (126 MHz, $CDCl_3$) δ 227.28, 133.84, 129.57, 128.49, 69.28, 14.39. ν_{max}/cm^{-1} 3042

(s, Aryl-H), 2990 (s, CH), 1477 (bend asymmetrical, CH), 1431 (s, P-Ph), 1362 (b, symmetrical CH), 1189 (s, C-O-C), 1138 (s, CO), 1090 (-O-CS-S-), 1039 (C=S).

Synthesis of tin(II)ethyl xanthate, [Sn(S_2COEt)$_2$]

The procedure used is adapted from the method of Raston *et al.*.[4] Potassium ethylxanthogenate (12.00 g, 0.075 mol) was stirred in dry methanol for 20 minutes and was subsequently cooled to 0 ˚C. $SnCl_2$ (7.10 g, 0.037 mol) in dry methanol was added slowly to the swirled potassium ethylxanthogenate solution resulting in the formation of a cream precipitate. The cream precipitate was collected by vacuum filtration and was washed with 100 ml of dry methanol previously cooled to 0 ˚C. The precipitate was dried *in vaceo* and a cream powder was collected (6.32 g, 49.6 %). Mp = 50-52 ˚C (Found: C, 19.70; H, 2.74; S, 34.66. Calc. for [Sn(S_2COEt)$_2$]: C, 19.96; H, 2.79; S, 35.52%) ^{1}H NMR (500 MHz, $CDCl_3$) δ 4.56 (2 H, q, CH_2), 1.50 (3 H, t, CH_3). ^{13}C NMR (126 MHz, $CDCl_3$) δ 220.98, 74.50, 14.19. ν_{max}/cm^{-1} 2988 (s, CH), 1462 (bend asymmetrical, CH), 1362 (b, symmetrical CH), 1190 (s, C-O-C), 1108 (s, CO), 1022 (C=S).

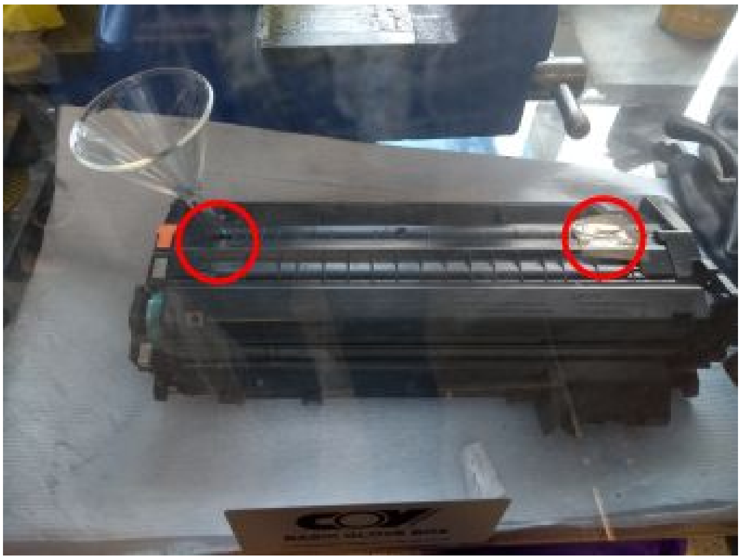

Figure S1. A Hewlett Packard CE505A toner cartridge that has had holes drilled at the locations shown by the red circles.

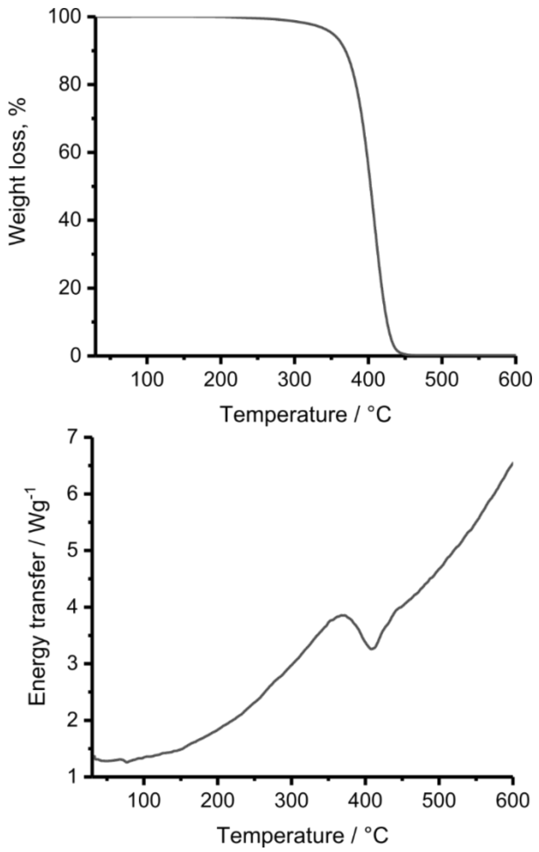

Figure S2. Thermal properties of poly(styrene-*co*-*n*-butyl acrylate) with (top) TGA profile and (bottom) DSC measurement.

Table S1. Mean polymer particle sizes for the commercial toner and the poly(styrene-*co*-*n*-butyl acrylate) metal xanthate mixtures.

	Mean particle width / µm	Standard deviation / µm
HP CE505A toner	7.52	2.15
Polymer and $[Zn(S_2COEt)_2]$	6.07	3.13
Polymer and $[Sn(S_2COEt)_2]$	4.27	4.75
Polymer and $[Cu(S_2COEt).(PPh_3)_2]$	4.46	4.64

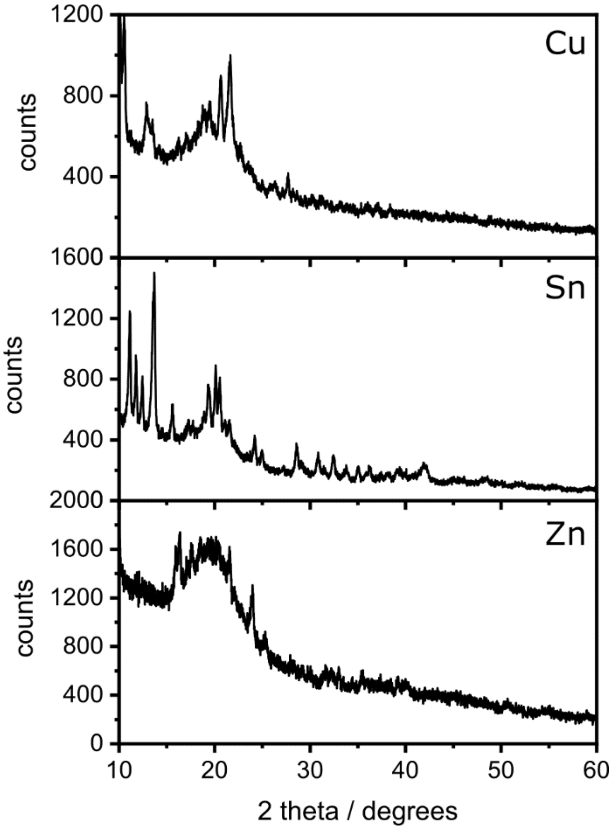

Figure S3. XRD diffractograms of ball-milled poly(styrene-*co*-*n*-butyl acrylate) with a metal xanthate complexes: [Cu(S_2COEt).(PPh_3)$_2$] (top), [Sn(S_2COEt)$_2$] (middle) and [Zn(S_2COEt)$_2$] (bottom).

Table S2. Ratio of elements unique to metal xanthates observed in milled particles from EDX spectra. Observed(Expected)

	Polymer and [Zn(S_2COEt)$_2$]	Polymer and [Sn(S_2COEt)$_2$]	Polymer and [Cu(S_2COEt).(PPh_3)$_2$]
Cu	-	-	1(1)
Zn	1(1)	-	-
Sn	-	1(1)	-
S	4.3(4)	3.3(4)	2.4(2)

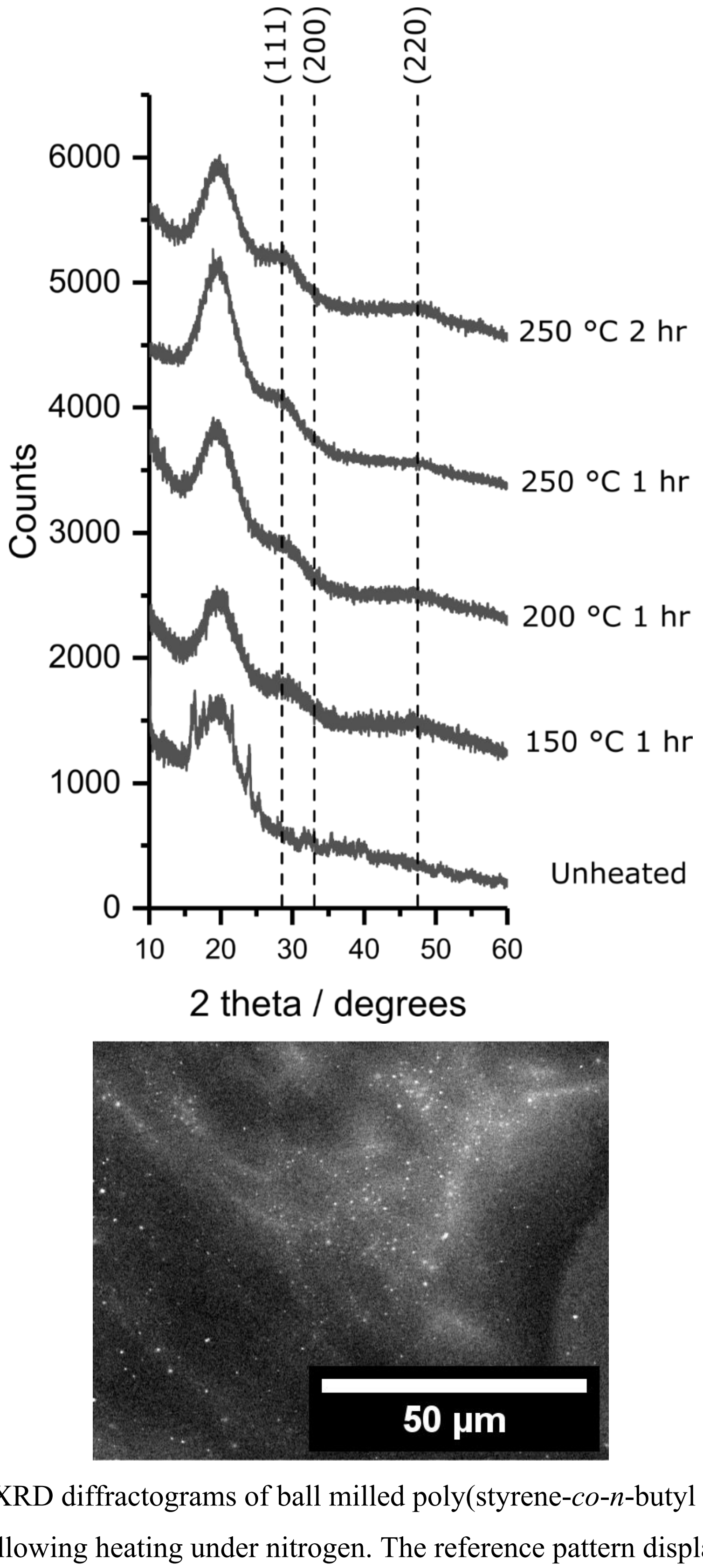

Figure S4. (top) XRD diffractograms of ball milled poly(styrene-*co*-*n*-butyl acrylate) with [$Zn(S_2COEt)_2$] following heating under nitrogen. The reference pattern displayed with dashed lines is for sphalerite ZnS ISCD #108733. (bottom) SEM micrograph of ZnS particles in poly(styrene-*co*-*n*-butyl acrylate) following heating at 250 °C for 2 hours.

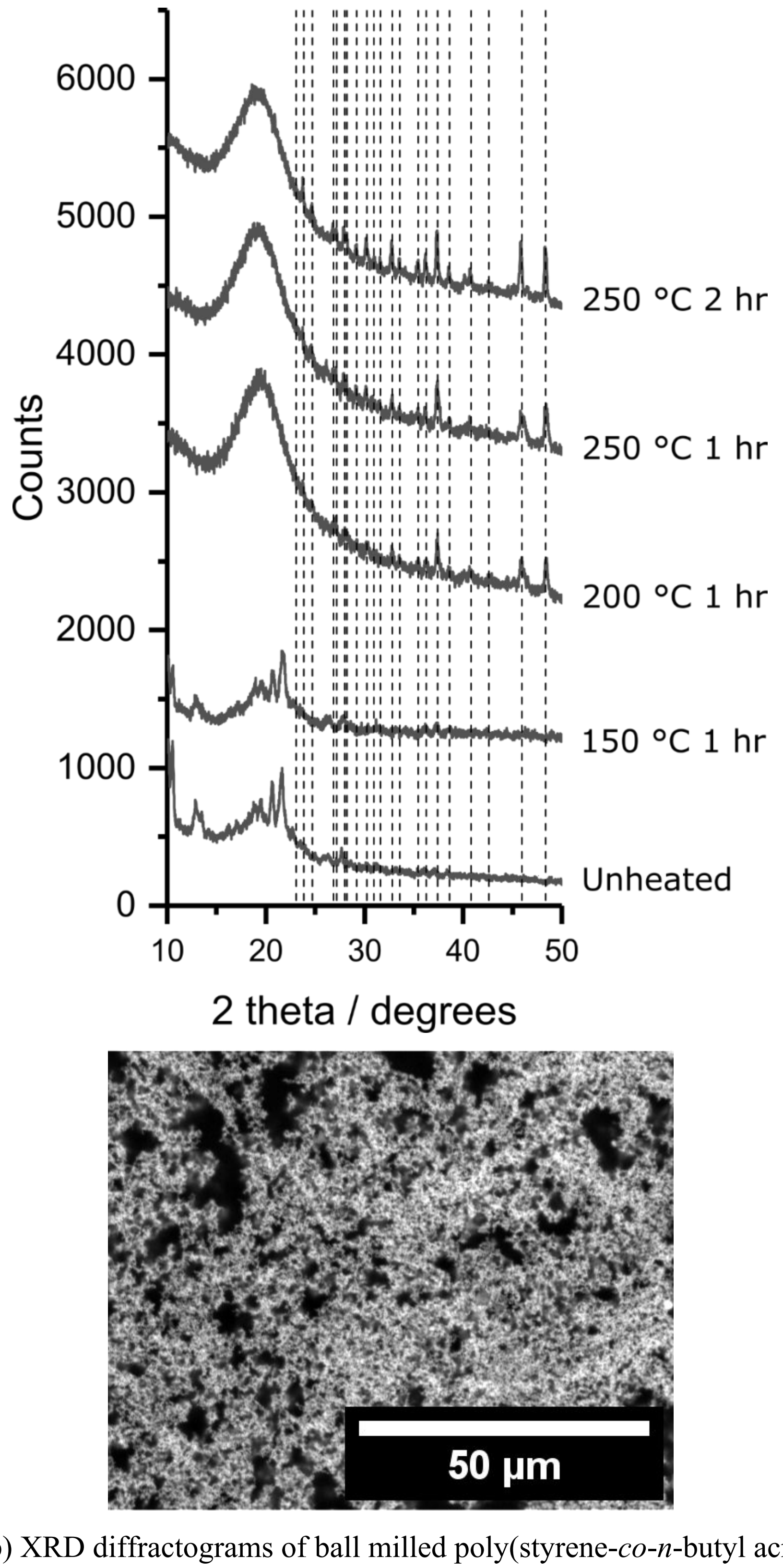

Figure S5. (top) XRD diffractograms of ball milled poly(styrene-*co*-*n*-butyl acrylate) with [Cu(S_2COEt).(PPh_3)$_2$] following heating under nitrogen. The reference pattern displayed with dashed lines is for chalcocite Cu_2S ISCD #106030. (bottom) SEM micrograph of Cu_2S particles in poly(styrene-*co*-*n*-butyl acrylate) following heating at 250 °C for 2 hours.

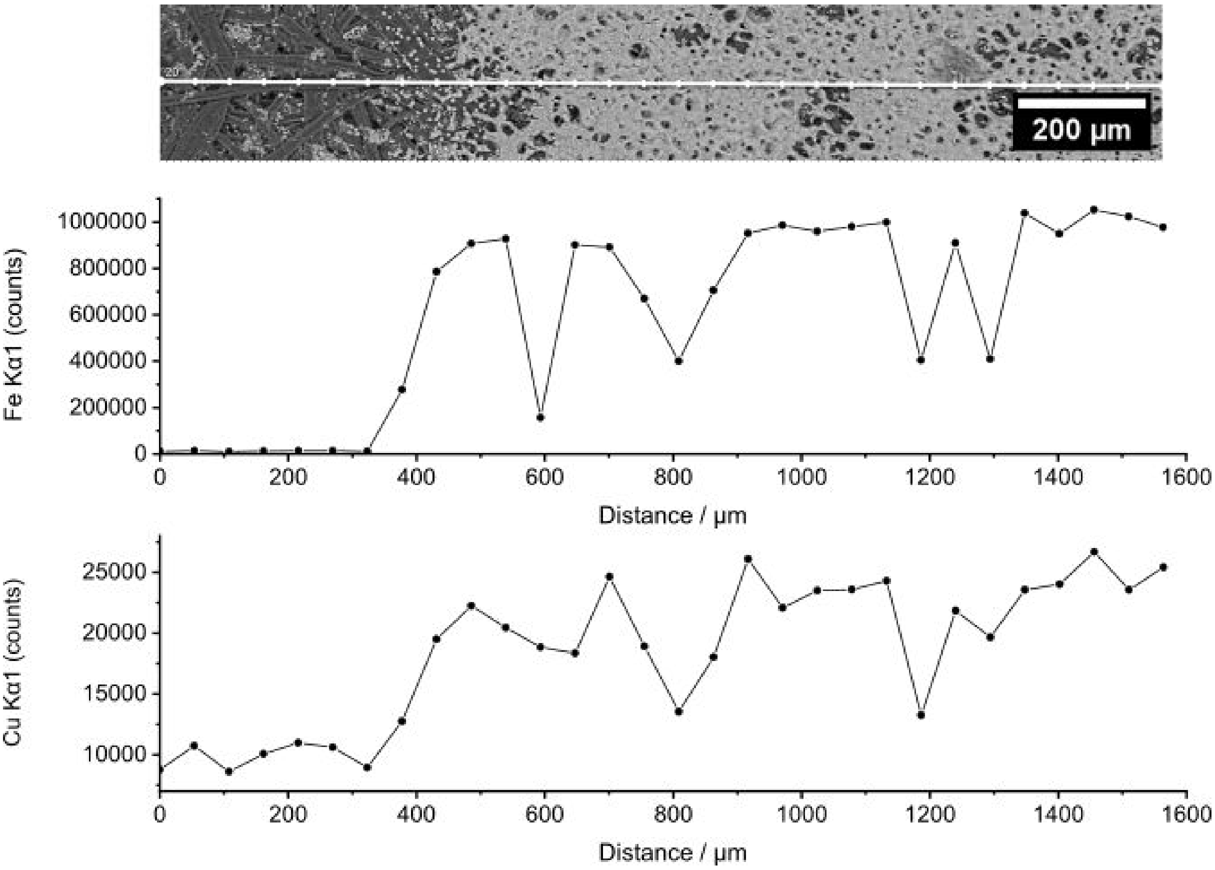

Figure S6. EDX line scans of printed [Cu(S_2COEt).(PPh_3)$_2$] containing poly(styrene-*co*-*n*-butyl acrylate) with HP toner where the column (top) SEM micrograph showing region measured, (middle) the line scan form the Fe Kα1 signal and (bottom) the line scan form the Cu Kα1 signal.

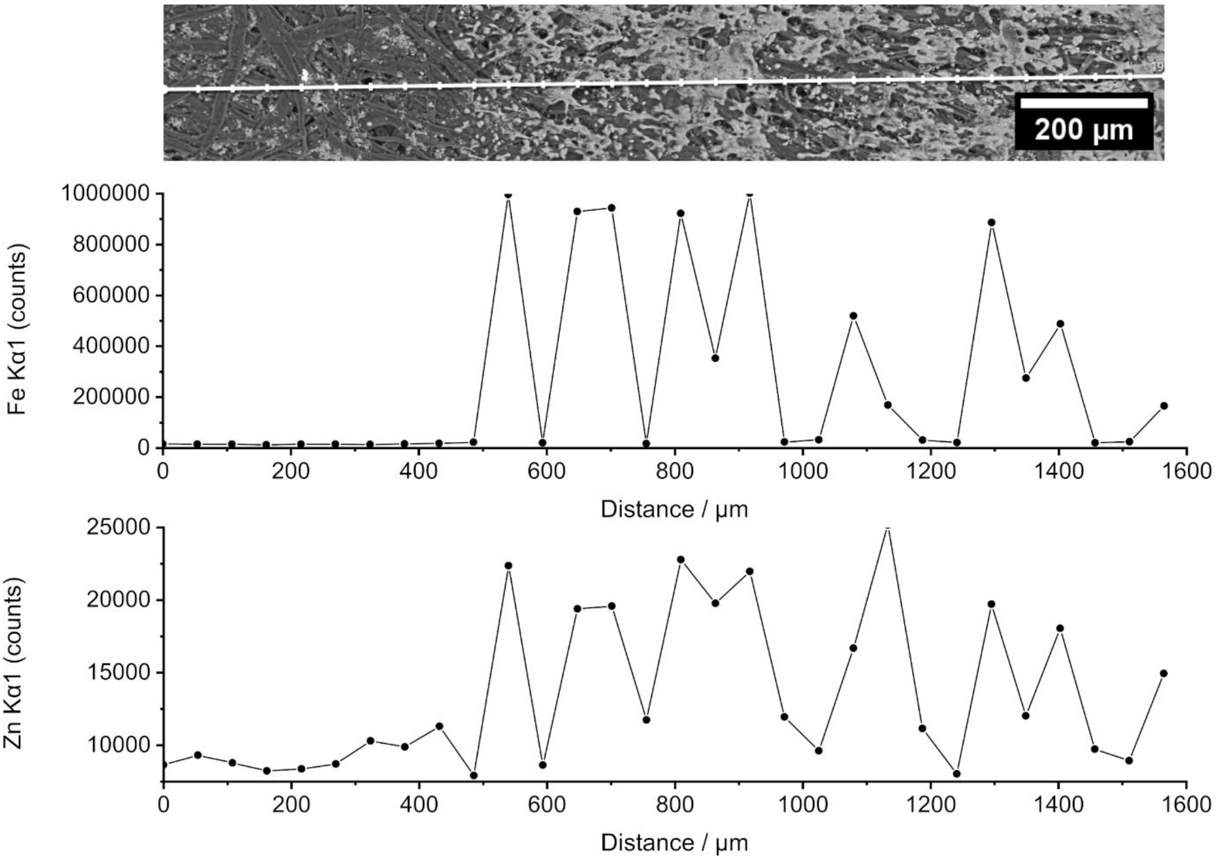

Figure S7. EDX line scans of printed [Zn(S_2COEt)$_2$] containing poly(styrene-*co*-*n*-butyl acrylate) with HP toner where the column (top) SEM micrograph showing region measured, (middle) the line scan form the Fe Kα1 signal and (bottom) the line scan form the Zn Kα1 signal.

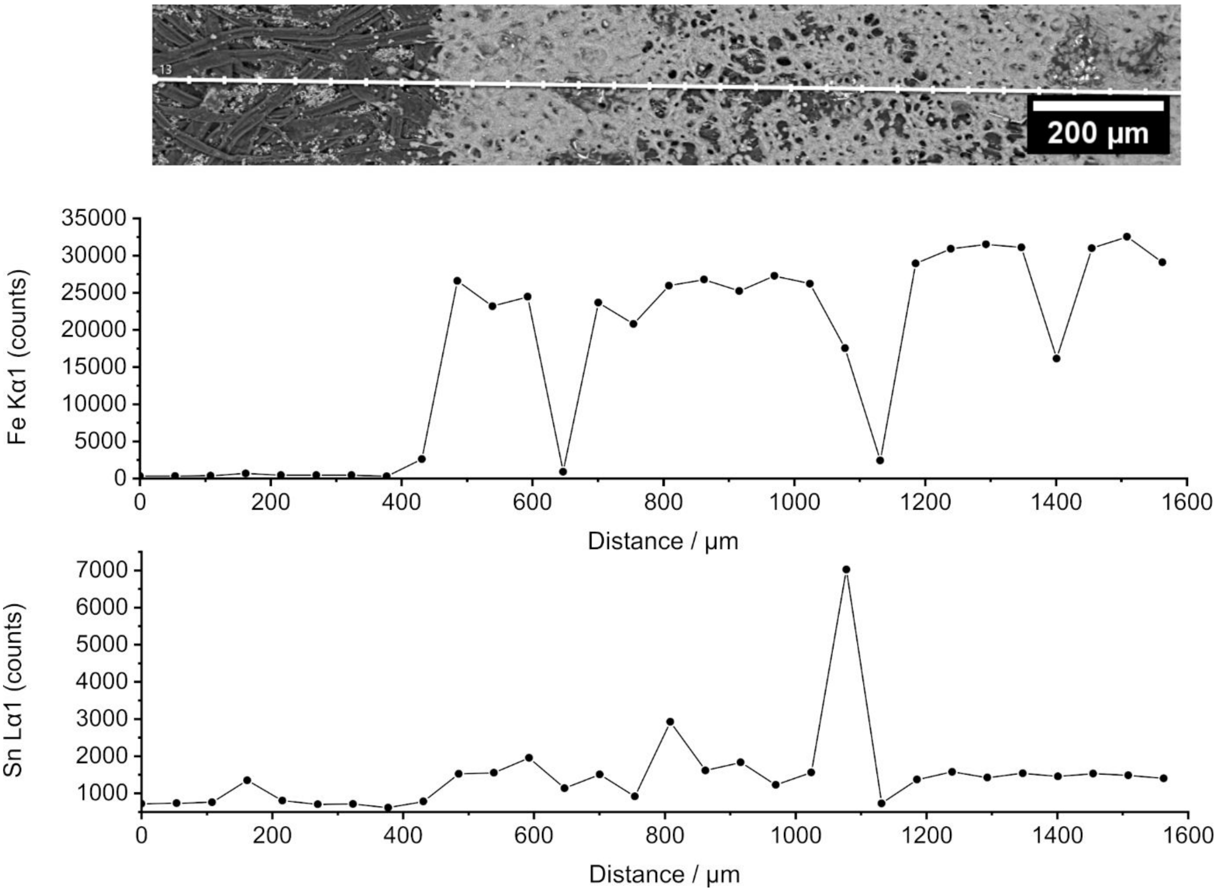

Figure S8. EDX line scans of printed [$Sn(S_2COEt)_2$] containing poly(styrene-*co*-*n*-butyl acrylate) with HP toner where the column (top) SEM micrograph showing region measured, (middle) the line scan form the Fe Kα1 signal and (bottom) the line scan form the Sn Lα1 signal.

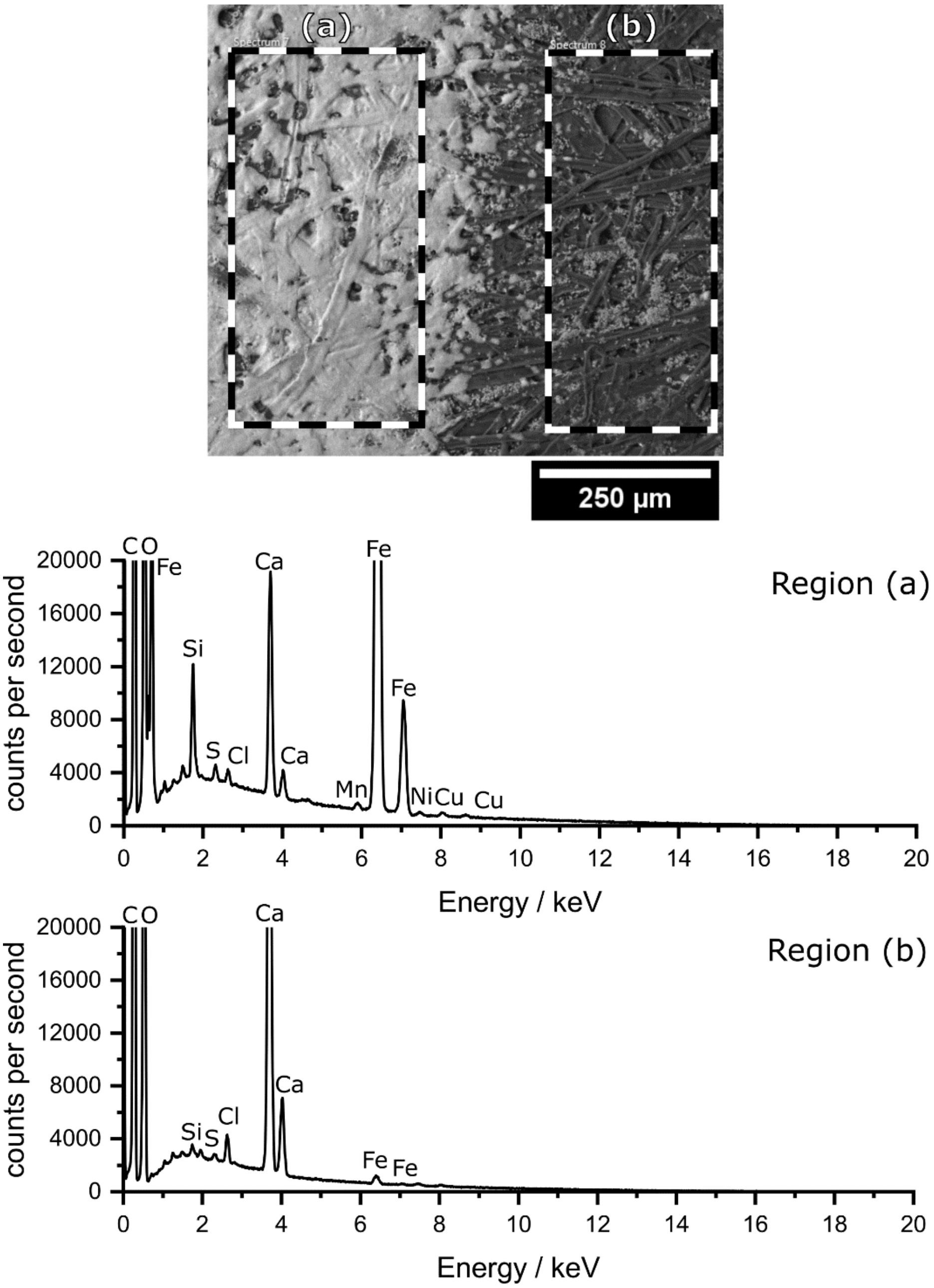

Figure S9. An SEM micrograph containing (a) printed and (b) unprinted regions with the corresponding EDX spectra beneath. The printed toner consisted of [Cu(S_2COEt).(PPh_3)$_2$] containing poly(styrene-*co*-butyl acrylate) printed with HP toner following heating to 250 °C for 2 hours.

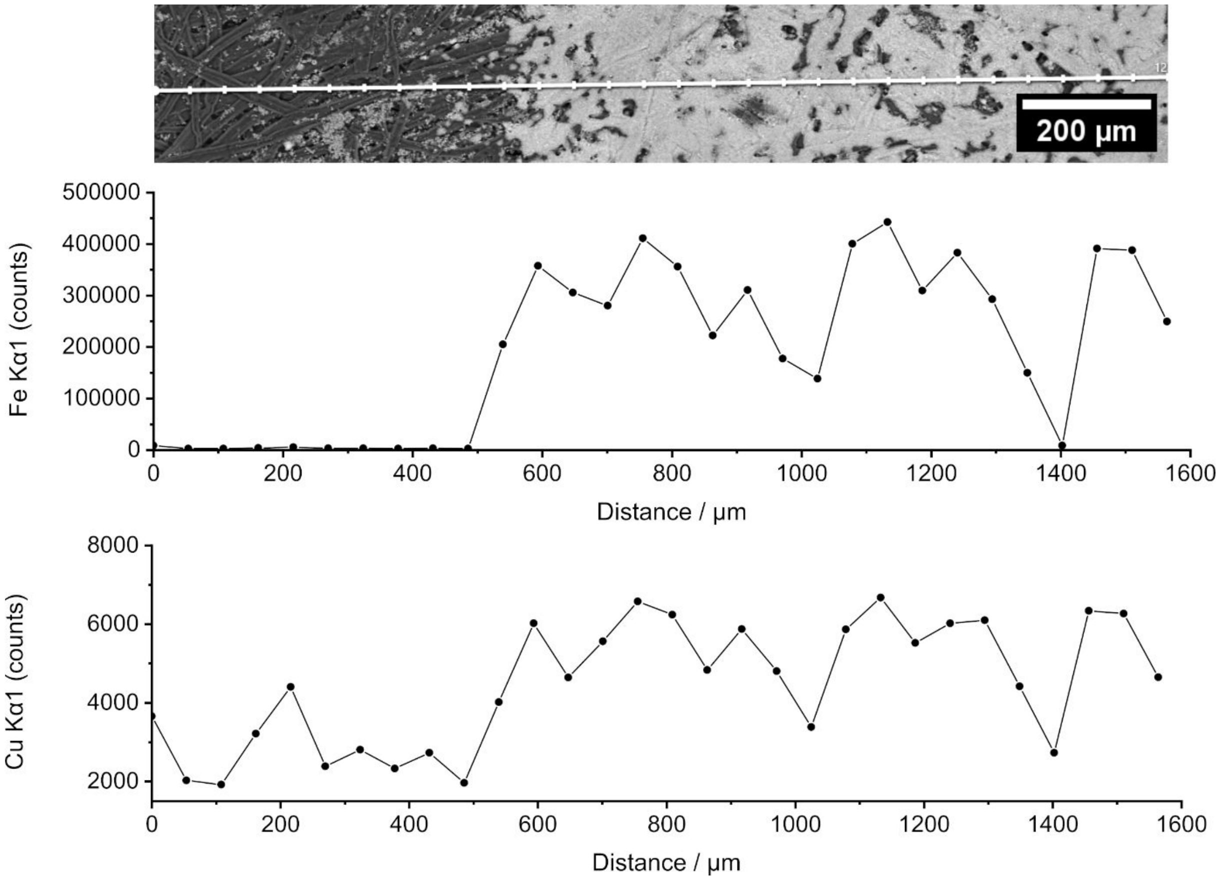

Figure S10. EDX line scans following heating at 250 °C for 2 hours of printed [$Cu(S_2COEt).(PPh_3)_2$] containing poly(styrene-*co*-*n*-butyl acrylate) with HP toner where the column (top) SEM micrograph showing region measured, (middle) the line scan form the Fe Kα1 signal and (bottom) the line scan form the Cu Kα1 signal.

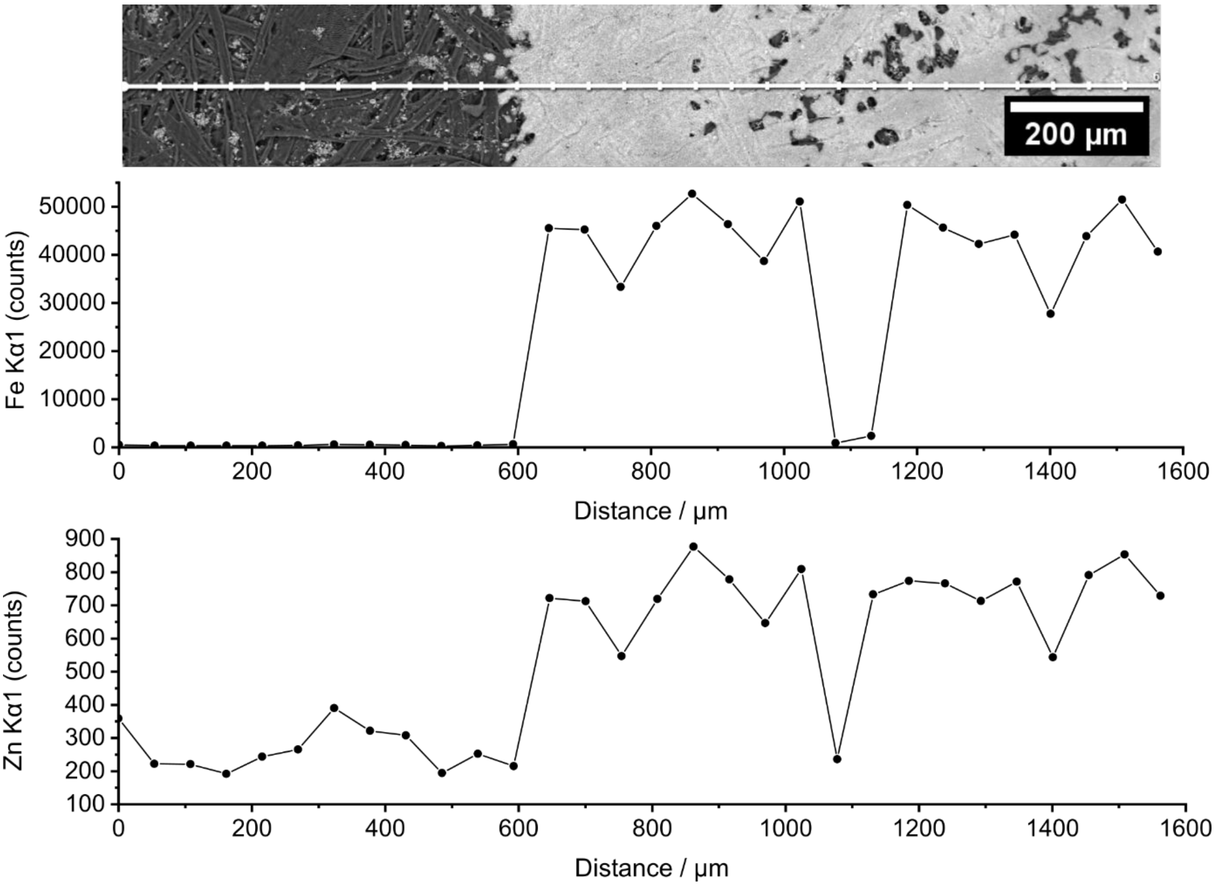

Figure S11. EDX line scans following heating at 250 °C for 2 hours of printed [$Zn(S_2COEt)_2$] containing poly(styrene-*co*-*n*-butyl acrylate) with HP toner where the column (top) SEM micrograph showing region measured, (middle) the line scan form the Fe Kα1 signal and (bottom) the line scan form the Zn Kα1 signal.

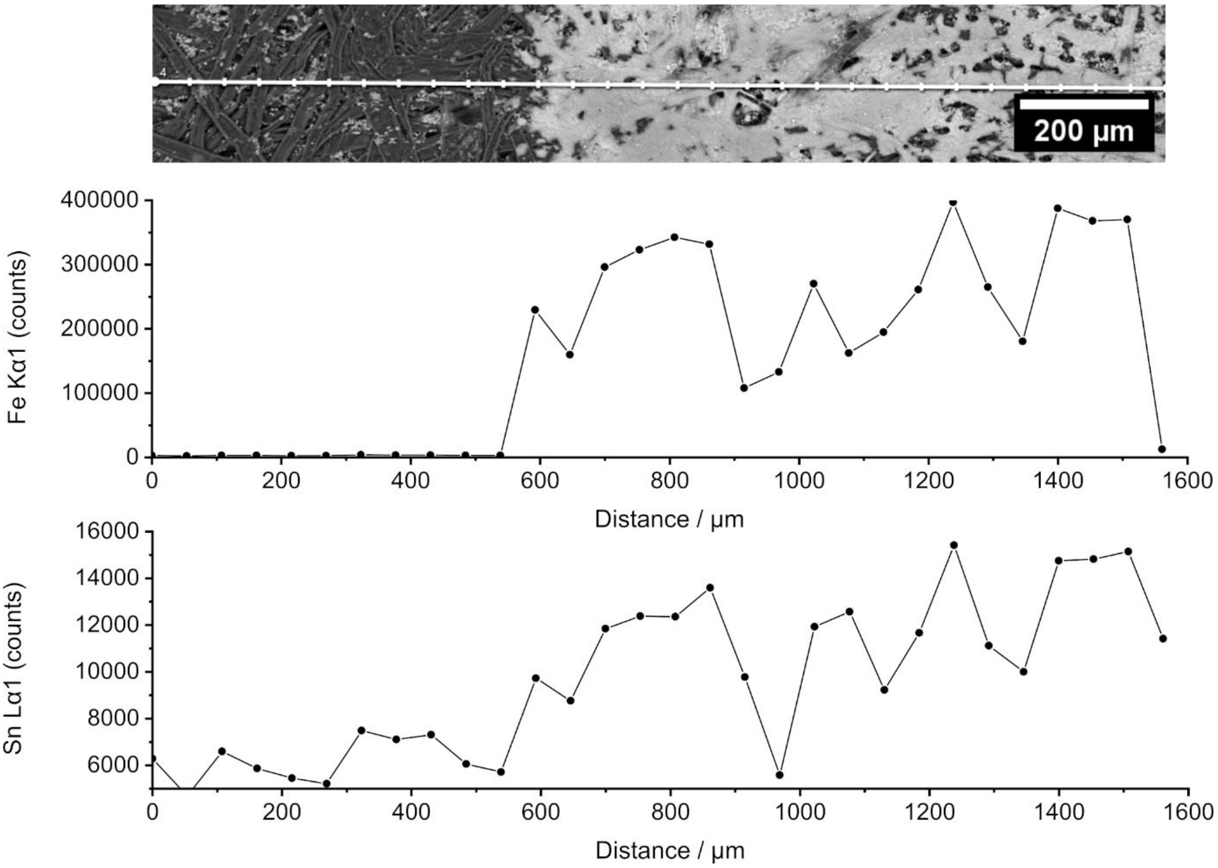

Figure S12. EDX line scans following heating at 250 °C for 2 hours of printed [Sn(S_2COEt)$_2$] containing poly(styrene-*co*-*n*-butyl acrylate) with HP toner where the column (top) SEM micrograph showing region measured, (middle) the line scan form the Fe Kα1 signal and (bottom) the line scan form the Sn Lα1 signal.

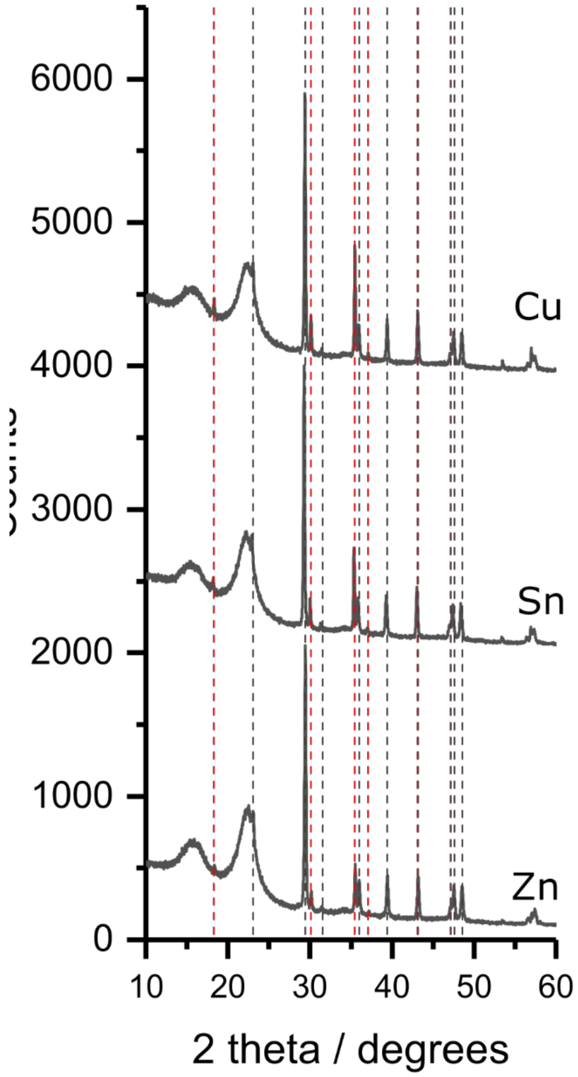

Figure S13. XRD diffractograms of paper printed with precursor containing toners following heating at 250 °C for 2 hours under nitrogen. The dashed lines represent the 2 theta values for $CaCO_3$ ISCD #18166 (black) and Fe_3O_4 ISCD #183969 (red).

Raman microscopy

The printed metal chalcogenides and the controls were examined by Raman microscopy in an effort to observe the metal chalcogenide phases by observation of their characteristic Raman peaks as previously reported.[5] The expected peaks metal chalcogenides are expected at 275 and 352 cm^{-1} for cubic ZnS;[6] at 160, 190 and 219 cm^{-1} for SnS;[7] and 264 and 475 cm^{-1} for $Cu_{2-x}S$.[8,9] Luminescence of poly(styrene-co-*n*-butyl acrylate) and the resulting metal chalcogenides , Figure S14, results in no observable Raman signals for the expected metal chalcogenides in the control experiments containing only these components, Figures S15 – S17. Raman spectra were collected for the metal xanthate containing toner after being printed and heated, Figures S18 to S20. These contained observable no Raman signals expected from the observed controls, Table S3 and Figure S14, nor the expected metal chalcogenides. This is despite the optical observation of inorganic material that matches the inorganic materials observed by SEM in morphology. It was concluded that the combination of absorption and luminescence profiles of components results in the inability to observe any Raman signals, including those previously observed in the controls.

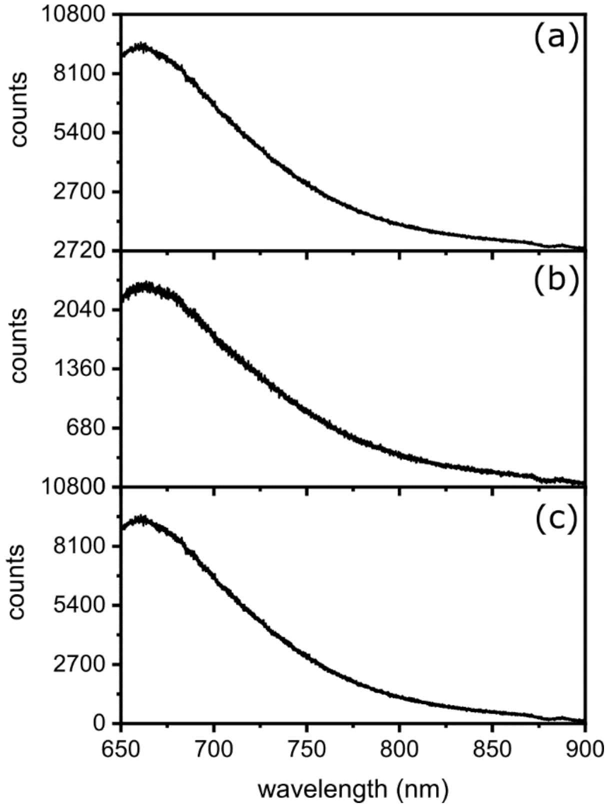

Figure S14. Luminescence spectra when excited at 633nm for (a) paper, (b) commercial toner and (c) poly(styrene-*co*-*n*-butyl acrylate) after heating at 250 °C for 2 hours.

Table S3. Observed Raman shifts of components contained in control spectra for toner mixture and paper when using incident light of 785 nm.

Component	Peak position / cm^{-1}	Assignments and/or description	Reference
Polymerised styrene in poly(styrene-*co*-*n*-butyl acrylate)	999.77	$\nu_1(A_1)$, $\nu'_{13}(A_1)$	10
	1031.99	$\nu_{18A}(A_1)$	10
Magnetite, Fe_3O_4 in commercial toner	666.55	A_{1g}	11
Polymerised styrene in commercial toner	999.72	$\nu_1(A_1)$, $\nu'_{13}(A_1)$ of polystyrene	10
$CaCO_3$	1083.60	ν_1 of CO_3^{2-} in calcite	12
cellulose	329.45<br>379.95<br>435.04<br>458.00<br>1089.28<br>1120.27	Characteristic Raman fingerprint of cellulose	13–15

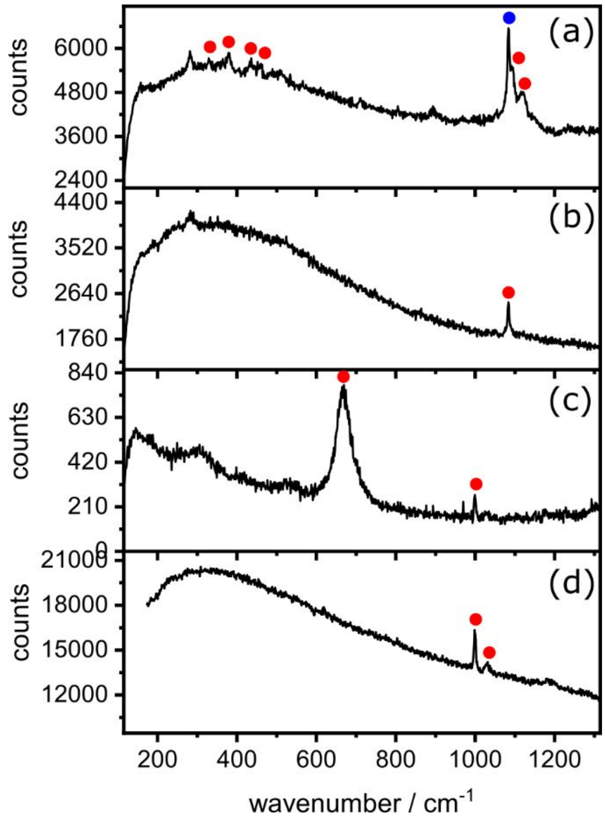

Figure S15. Raman spectra collected with incident light of 785 nm for (a) a cellulose rich area of printer paper, (b) a $CaCO_3$ rich area of printer paper, (c) printed commercial toner and (d) poly(styrene-*co*-*n*-butyl acrylate) heated to 250 °C for 2 hours. The red dots correspond to the values listed in Table S3. The blue dot is $CaCO_3$ in the cellulose rich area.

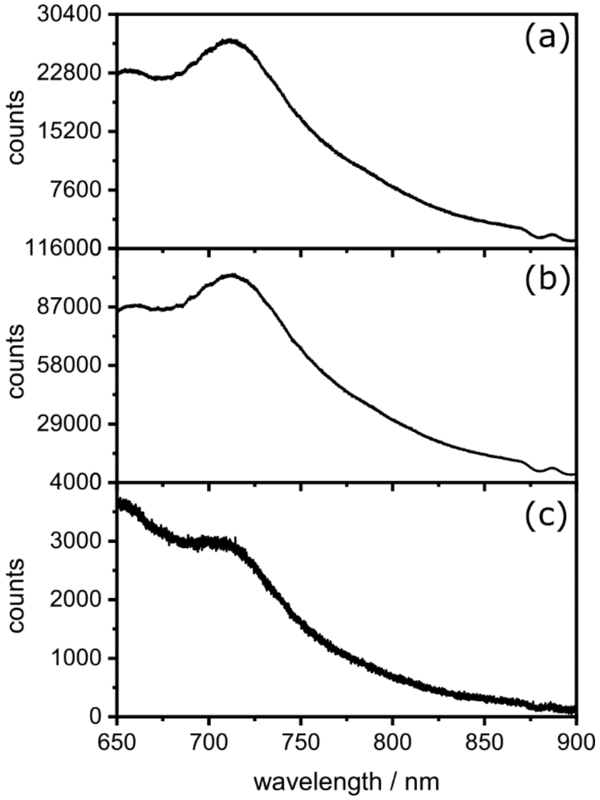

Figure S16. Luminescence spectra from milled mixtures of poly(styrene-*co*-*n*-butyl acrylate) with metal xanthate complexes after heating at 250 °C for 2 hours. The polymer was mixed with (a) [Cu(S_2COEt).(PPh_3)$_2$], (b) [Sn(S_2COEt)$_2$] or (c) [Zn(S_2COEt)$_2$] prior to heating.

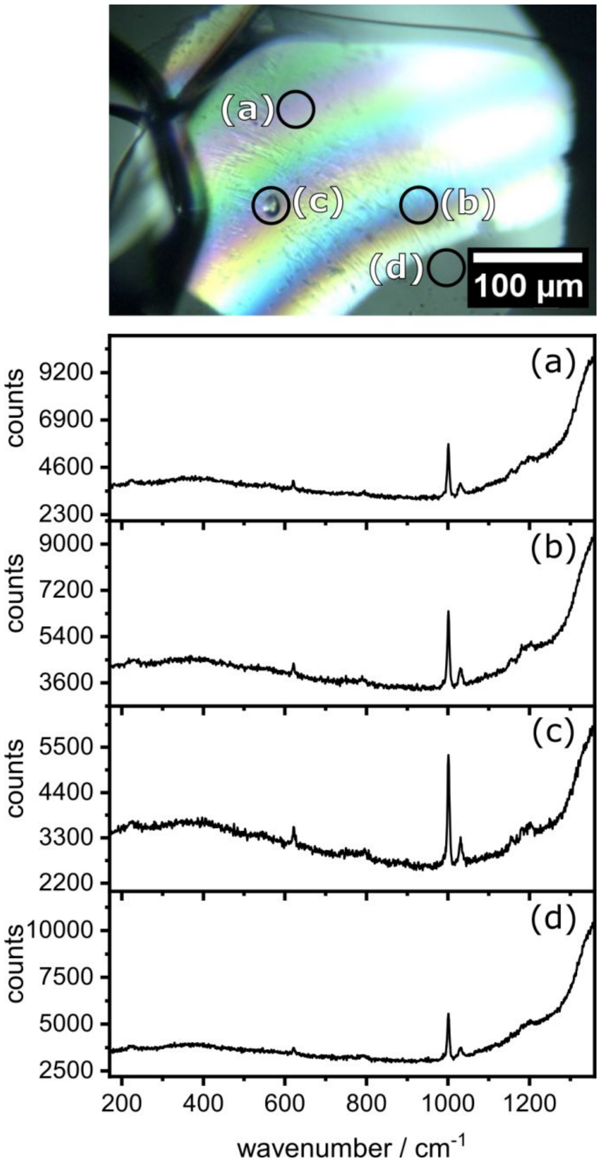

Figure S17. Optical micrograph and corresponding point Raman spectra for milled $[Zn(S_2COEt)_2]$ and poly(styrene-*co*-*n*-butyl acrylate) after heating at 250 °C for 2 hours.The observed signal corresponds to the polystyrene component of poly(styrene-*co*-*n*-butyl acrylate).

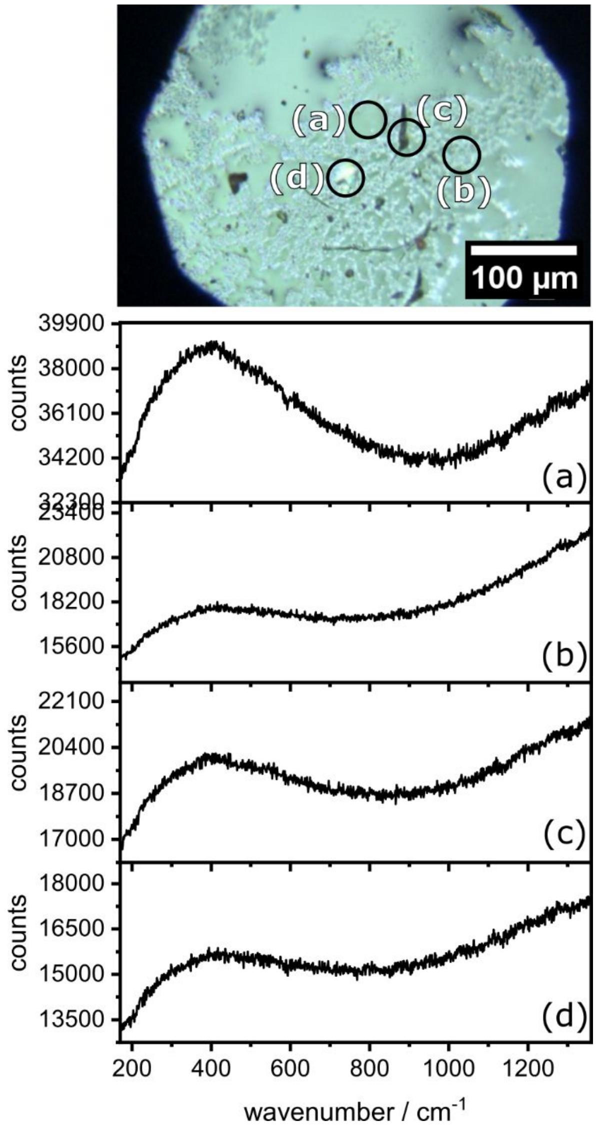

Figure S18. Optical micrograph and corresponding point Raman spectra for milled[$Cu(S_2COEt).(PPh_3)_2$] and poly(styrene-*co*-*n*-butyl acrylate) after heating at 250 °C for 2 hours.

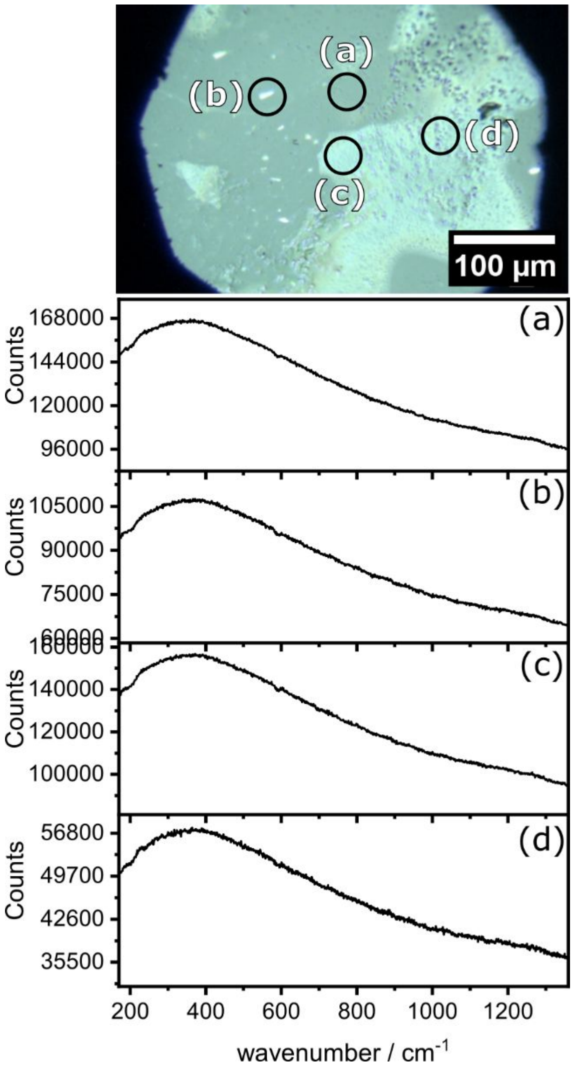

Figure S19. Optical micrograph and corresponding point Raman spectra for milled [Sn(S_2COEt)$_2$] and poly(styrene-*co*-*n*-butyl acrylate) after heating at 250 °C for 2 hours.

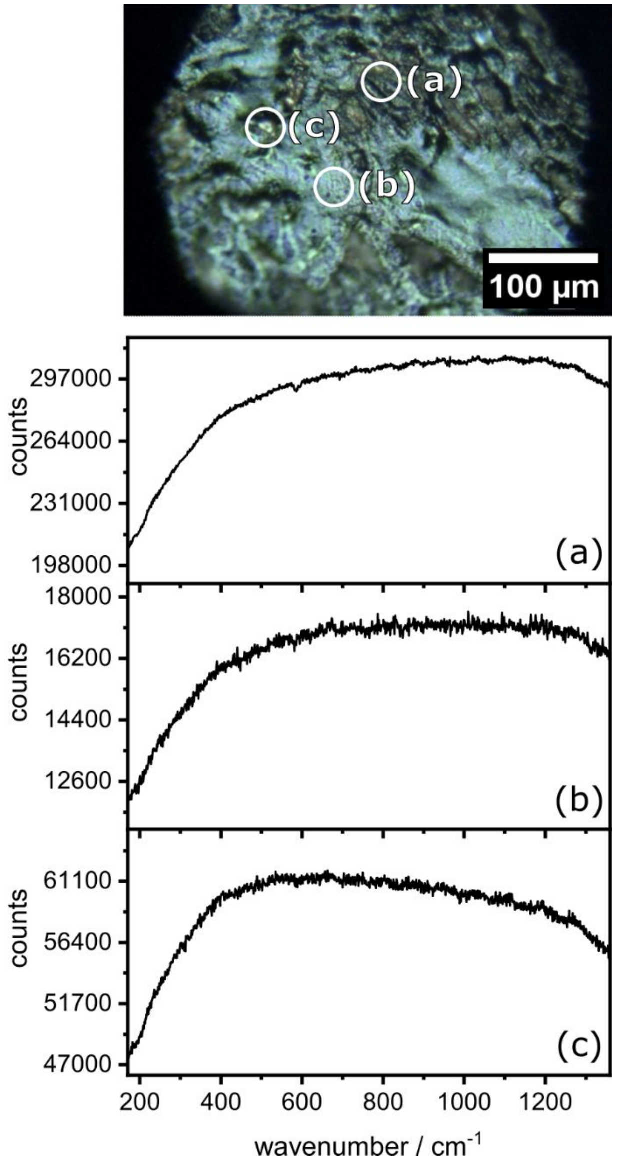

Figure S20. Optical micrograph and corresponding point Raman spectra for printed $[Zn(S_2COEt)_2]$ containing poly(styrene-*co*-*n*-butyl acrylate) with HP toner after heating at 250 °C for 2 hours.

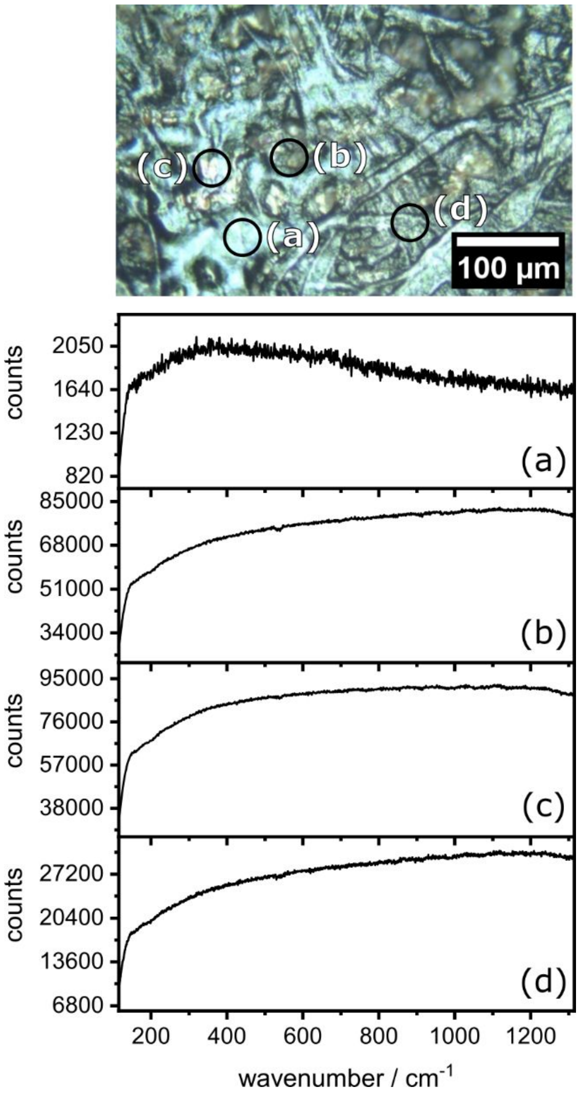

Figure S21. Optical micrograph and corresponding point Raman spectra for printed [$Cu(S_2COEt).(PPh_3)_2$] containing poly(styrene-*co*-*n*-butyl acrylate) with HP toner after heating at 250 °C for 2 hours.

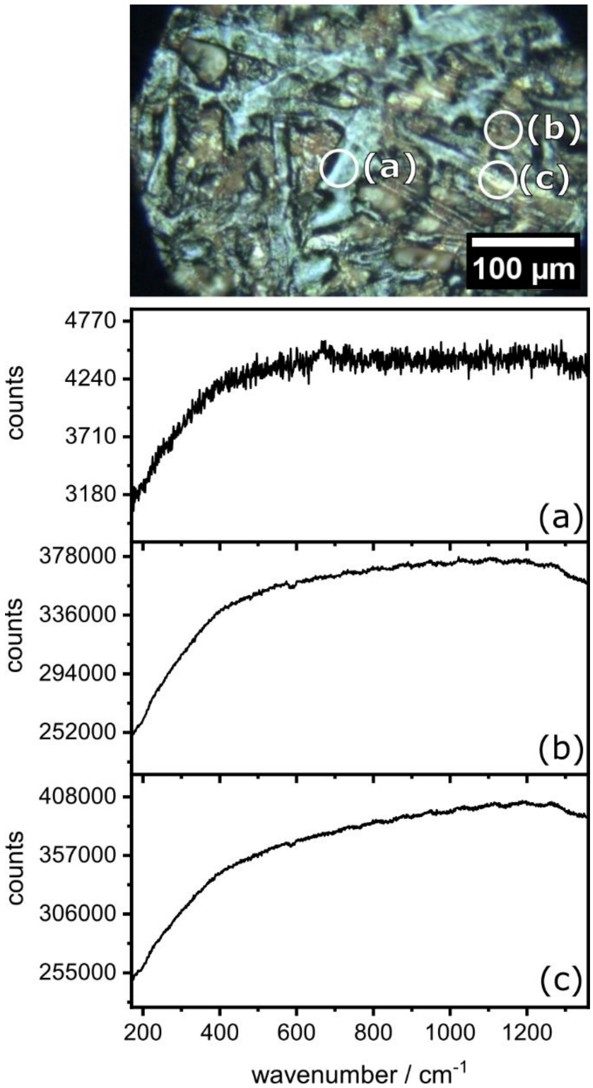

Figure S22. Optical micrograph and corresponding point Raman spectra for printed [$Sn(S_2COEt)_2$] containing poly(styrene-*co*-*n*-butyl acrylate) with HP toner after heating at 250 °C for 2 hours.

References

(1) Kociok-Köhn, G.; Molloy, K. C.; Sudlow, A. L. Molecular Routes to Cu_2ZnSnS_4: A Comparison of Approaches to Bulk and Thin-Film Materials. *Can. J. Chem.* **2014**, *92* (6), 514–524. https://doi.org/10.1139/cjc-2013-0497.

(2) Cariati, F.; Ganadu, M. L.; Naldini, L.; Seneci, S. *O*-Alkyldithiocarbonato Triphenylphosphine Complexes of IB Metals. *Gazz. Chim. Ital.* **1979**, *109*, 181–185.

(3) Kumar, A.; Mayer-Figge, H.; Sheldrick, W. S.; Singh, N. Synthesis, Structure, Conductivity, and Calculated Nonlinear Optical Properties of Two Novel Bis(Triphenylphosphane)Copper(I) Dithiocarbamates. *Eur. J. Inorg. Chem.* **2009**, *2009* (18), 2720–2725. https://doi.org/10.1002/ejic.200900013.

(4) Raston, C. L.; Tennant, P. R.; White, A. H.; Winter, G. Reactions of Tin(II) and Tin(IV) Xanthates: Crystal Structure of Tetrakis(*O*-Ethylxanthato)Tin(IV). *Aust. J. Chem.* **1978**, *31* (7), 1493–1500. https://doi.org/10.1071/ch9781493.

(5) Fernandes, P. A.; Salomé, P. M. P.; da Cunha, A. F. Study of Polycrystalline Cu_2ZnSnS_4 Films by Raman Scattering. *J. Alloy. Compd.* **2011**, *509* (28), 7600–7606. https://doi.org/10.1016/j.jallcom.2011.04.097.
(6) Nilsen, W. G. Raman Spectrum of Cubic ZnS. *Phys. Rev.* **1969**, *182* (3), 838–850. https://doi.org/10.1103/PhysRev.182.838.
(7) Price, L. S.; Parkin, I. P.; Hardy, A. M. E.; Clark, R. J. H.; Hibbert, T. G.; Molloy, K. C. Atmospheric Pressure Chemical Vapor Deposition of Tin Sulfides (SnS, $Sn_2S_{,3}$, and SnS_2) on Glass. *Chem. Mater.* **1999**, *11* (7), 1792–1799. https://doi.org/10.1021/cm990005z.
(8) Mernagh, T. P.; Trudu, A. G. A Laser Raman Microprobe Study of Some Geologically Important Sulphide Minerals. *Chem. Geol.* **1993**, *103* (1), 113–127. https://doi.org/10.1016/0009-2541(93)90295-T.
(9) Munce, C. G.; Parker, G. K.; Holt, S. A.; Hope, G. A. A Raman Spectroelectrochemical Investigation of Chemical Bath Deposited Cu_xS Thin Films and Their Modification. *Colloid. Surface. A* **2007**, *295* (1), 152–158. https://doi.org/10.1016/j.colsurfa.2006.08.045.
(10) Liang, C. Y.; Krimm, S. Infrared Spectra of High Polymers. VI. Polystyrene. *J. Polym. Sci.* **1958**, *27* (115), 241–254. https://doi.org/10.1002/pol.1958.1202711520.
(11) Soler, M. A. G.; Qu, F. Raman Spectroscopy of Iron Oxide Nanoparticles. In *Raman Spectroscopy for Nanomaterials Characterization*; Kumar, C. S. S. R., Ed.; Springer: Berlin, Heidelberg, 2012; pp 379–416. https://doi.org/10.1007/978-3-642-20620-7_14.
(12) Behrens, G.; Kuhn, L. T.; Ubic, R.; Heuer, A. H. Raman Spectra of Vateritic Calcium Carbonate. *Spectrosc. Lett.* **1995**, *28* (6), 983–995. https://doi.org/10.1080/00387019508009934.
(13) Agarwal, U. P. 1064 Nm FT-Raman Spectroscopy for Investigations of Plant Cell Walls and Other Biomass Materials. *Front. Plant Sci.* **2014**, *5*.
(14) Agarwal, U. P. Analysis of Cellulose and Lignocellulose Materials by Raman Spectroscopy: A Review of the Current Status. *Molecules* **2019**, *24* (9), 1659. https://doi.org/10.3390/molecules24091659.
(15) Agarwal, U. P.; Ralph, S. A. FT-Raman Spectroscopy of Wood: Identifying Contributions of Lignin and Carbohydrate Polymers in the Spectrum of Black Spruce (Picea Mariana). *Appl. Spectrosc.* **1997**, *51* (11), 1648–1655.
