# Supplementary figures and images for: Semiconductor Deposition via Laser Printing of a Bespoke Toner Containing Metal Xanthate Complexes
Source: ACS Appl Eng Mater. 2024 May 8;2(5):1225–33. doi: 10.1021/acsaenm.3c00709 (PMC11129185; doi:10.1021/acsaenm.3c00709)

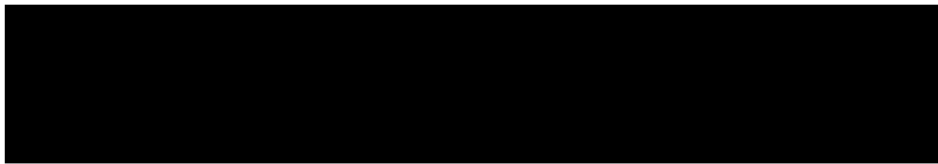

TEST ■ TEST

■ TEST ■

TEST ■ TEST

■ TEST ■

TEST ■ TEST

■ TEST ■

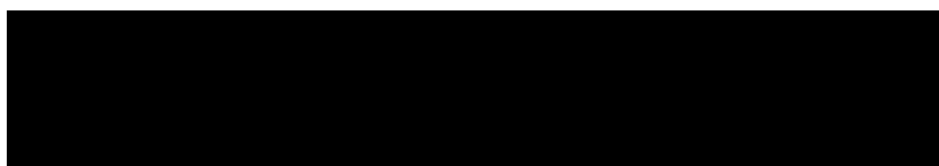

Supplement: Supplementary file 2 — em3c00709_si_002.pdf [file em3c00709_si_002.pdf]
